# Characterization of key aroma compounds in a novel Chinese rice wine *Xijiao Huojiu* during its biological-ageing-like process by untargeted metabolomics

**DOI:** 10.1016/j.heliyon.2024.e34396

**Published:** 2024-07-10

**Authors:** Han Wang, Rui Shang, Suying Gao, Ancheng Huang, Honghui Huang, Wenyang Li, Hongwei Guo

**Affiliations:** a Institute of Plant and Food Science, Department of Biology, School of Life Sciences, Southern University of Science and Technology (SUSTech), Shenzhen, 518055, China; b Key Laboratory of Molecular Design for Plant Cell Factory of Guangdong Higher Education Institutes, School of Life Sciences, Southern University of Science and Technology (SUSTech), Shenzhen, 518055, China; c Shenzhen Haohao Biotechnology Company Ltd., Shenzhen, 518028, China

**Keywords:** Chinese rice wine (CRW), Biological-ageing-like process, Headspace solid-phase microextraction (HS-SPME), Orthogonal partial least squares-discriminant analysis (OPLS-DA), Cocoa- and caramel-like aroma

## Abstract

Xijiao Huojiu (Xijiao), an ancient Chinese rice wine (ACRW), is produced using traditional methods, which involve biological-ageing-like process and result in distinctive sensory profiles. However, its aroma composition is still unclear. In this study, the aroma characteristics of three samples with varying ageing times were examined. Xijiao_SCT, with a short cellar time, exhibited a strong fruity and floral aroma and a less grain-like aroma. Conversely, Xijiao_LCT, which had a long cellar time, had a deep cocoa- and caramel-like aroma. A total of 27 key odorants that greatly influenced the aroma characteristics of Xijiao were identified. Comparative studies were used to identify 12 key odorants that distinguish Xijiao from modern Chinese rice wine (MCRW) and grape wines (GW). Additionally, 13 dominant latent ageing markers differentiated Xijiao_SCT from Xijiao_LCT. Our results suggested that ACRW and MCRW have overlapping but distinct volatile metabolomic profiles, highlighting the characteristics of ACRW during ageing process.

## Highlights

• The aroma characteristics of an ancient Chinese rice wine (*Xijiao Huojiu*) were evaluated through qualitative and quantitative approaches.
• Xijiao_SCT has fruity/floral aromas, while Xijiao_LCT has cocoa/caramel aromas.
• Twelve odorants were identified as potential discriminant compounds causing aroma differences among Xijiao, modern Chinese rice wines, and grape wines.
• Thirteen odorants were determined to be latent ageing markers of Xijiao.
• Compounds that presumably contributed to the cocoa flavor were also identified.

## 1 Introduction

Chinese rice wine (CRW), also known as huangjiu, is one of the three oldest drinks in the world, along with beer and grape wine. Its history can be traced back to 7000 BCE, as found in a previous study on a prehistoric fermented beverage from the early Neolithic village of Jiahu in China [1]. CRW is widely consumed in southern China because of its unique flavor, mild taste, low alcohol content, and abundant nutrients [2]. The sensory properties of aroma are considered as its most important attributes and are closely linked to consumer perception and acceptance [3]. Currently, there are three types of CRW based on the aroma profile: traditional aroma CRW, light aroma CRW, and special aroma CRW [4]. However, with improvements in living standards, consumers are increasingly demanding higher quality and greater variety of CRW, which is promoting innovation in the CRW industry. Therefore, optimizing and innovating aromatic features may be a tailored and viable approach.

**Fig. 1:**
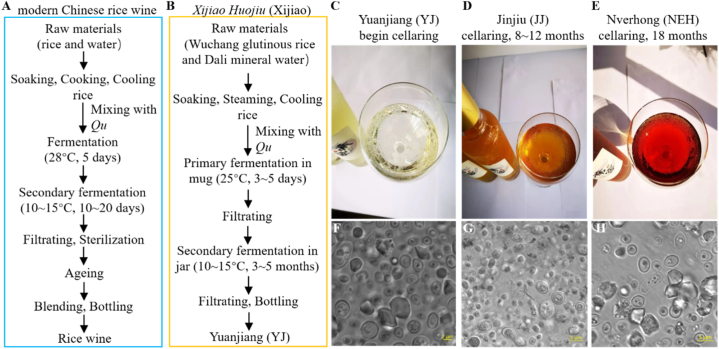
The production flow of making Xijiao Huojiu. (A) The brewing process of modern Chinese rice wine. (B) Schematic representation of the Xijiao production process. (C–E) Coloration and pigmentation of Xijiao after bottling. (F–H) Microbiomes enriched in Xijiao wines which were visualized under a microscope using 40 × object.

The overall flavor profile of CRW is influenced by the raw materials and production methods. Modern CRW (MCRW) is primarily made from rice or glutinous rice, using *Qu* as the fermentation agent. It goes through steps such as pre-fermentation, post-fermentation, pressing, filtration, sterilization, ageing, and secondary sterilization before being bottled (Fig. 1A). Over the past few decades, researchers have characterized volatile compounds of MCRW and identified key odorants that contribute to its aroma using techniques like gas chromatography-mass spectrometry (GC-MS), two-dimensional gas chromatography-mass spectrometry (GC × GC-MS), gas chromatography-olfactometry (GC–O), gas chromatography-ion mobility spectroscopy (GC-IMS), and low field-nuclear magnetic resonance (LF-NMR) [5,6]. The extraction of volatile compounds in wines has been a challenge due to the large number and complex performance of these compounds. Previous extraction approaches, including liquid-liquid extraction (LLE), supercritical fluid extraction (SFE), and solid-phase extraction (SPE), have limitations such as being time-consuming, low extraction efficiency, and solvent residue [6]. To overcome these shortcomings, cutting-edge techniques such as in-tube extraction (ITEX), solvent-free microwave extraction (SFME), vacuum distillation extraction (VDE) are recently developed and employed to recover trace substances with very low detection limits [7]. The headspace solid-phase microextraction (HS-SPME) has been widely used to extract and enrich volatile components, offering advantages such as high efficiency, sensitivity, and safety [7,8]. Through HS-SPME-GC-MS, researchers have explored flavor changes in CRW from the Shaoxing region during fermentation and the key aromatic components of CRW with varying total sugar contents [9,10]. Currently, MCRW contains over 680 aromatic compounds, including alcohols, esters, acids, aldehydes, ketones, phenols, nitrogen compounds, sulfur compounds, and hydrocarbons [11].

In archaic China, ancient CRW (ACRW) is produced using a traditional brewing method that skips sterilization before bottling. One notable example of ACRW is Nverhong, which was first described in the book *Nanfang Caomu Zhuang* (Herbs and Trees in the South of China), written in 304 CE by Han Ji, a famous writer and botanist in the Western Jin Period [12]. During an 18-year cellular stage, Nverhong experiences ongoing effects from both live microbes and dead cells. As a result, a significant amount of microbial activity occurs within pottery jars, which is believed to greatly impact the aroma characteristics of the CRW [13]. In the process of making grape wine (GW), the grape mash undergoes a long biological ageing process in barrels, aided by a complex microbial consortium consisting of non-*Saccharomyces* yeast species and *Saccharomyces cerevisiae* [14,15]. This microbial community plays a crucial role in transforming the characteristics of the wine. In traditional sparkling wine production, the wine undergoes changes as a result of its contact with yeast lees, predominantly composed of dead yeasts, following the second fermentation in sealed bottles [16,17]. This interaction with live microbes and their deceased cells within the wine is indicative of their substantial impact on the wine's aroma profile. Therefore, ACRW undergoes a biological-ageing-like process similar to some extent of GW. Thus, ACRW will likely exhibit distinct aroma properties compared to MCRW. However, the aromatic features of ACRW are still unclear.

*Xijiao Huojiu* (Xijiao) is a traditional Hakka rice wine from Guangdong Province, in southern China that preserves ancient brewing craftsmanship. Xijiao is known as “Huojiu” which means that living microbiomes remain in the rice wine and biologically control the ageing process. It is made using glutinous rice, mineral water, and ancestral *Qu* as fermentation agents (Fig. 1B). The duration of post-fermentation in bottles determines the three main types of Xijiao (Fig. 1C–E): Yuanjiang (YJ, beginner), Jinjiu (JJ, approximately 1 year), and Nverhong (NEH, more than 1.5 years). The presence of numerous bubbles in Xijiao indicates fermentation and suggests vigorous microbial activity in the bottle (Fig. 1C–E). The mash was bottled without sterilization after secondary fermentation, preserving numerous microbes in Xijiao (Fig. 1F–H). This made the bottle a fermentation container like a pottery jar, providing a place for further fermentation. Furthermore, Xijiao differentiates itself from MCRW by using oak, commonly used in the GW industry, in its closure. The choice of closure has been linked to aroma formation in GW [18]. These distinctive aspects of Xijiao's production process and packaging materials lead to a conceivable difference in its aroma profile compared to MCRW. Notably, consumers' feedback suggests that Xijiao, with a short cellar time, has a strong flavor of fruits and flowers, resembling GW. Therefore, it would be interesting to reveal the potential similarities between the aroma profiles of Xijiao and GW. In addition, characterizing unknown aromatic compounds in Xijiao will contribute to uncovering the flavor traits of ACRW.

In this study, we conducted a quantitative descriptive analysis (QDA) of sensory evaluations to gain a preliminary understanding of Xijiao and other fermented beverages (MCRW and GW). Next, untargeted GC-MS analysis was performed to identify the aroma compounds in all samples. This analysis allowed us to better understand the flavor differences between Xijiao and other wines, as well as the changes in aroma over different ageing time. Additionally, the calculation of odor activity values (OAVs) provided insights into the importance of individual aroma compounds for the sensory characteristics of each sample. Furthermore, through multivariate statistical analysis, we identified the key odorants that distinguished Xijiao from other wines. Orthogonal partial least-squares discriminant analysis (OPLS-DA) was used to identify the ageing markers of Xijiao. Finally, our study provided a fundamental understanding of the aroma profile of ACRW. In summary, studying Xijiao complements CRW and offers the potential to inject new vitality into the CRW market while providing theoretical support for the development of novel CRW.

## 2 Materials and methods

### 2.1 Chemicals and reagents

All chemicals and reagents were of chromatography grade (≥97 % purity) and ordered from Sigma-Aldrich Co. (Shanghai, China), including acetic acid, octanoic acid, decanoic acid, hexadecanoic acid, 1-propanol, isobutanol, isoamyl alcohol, phenylethyl alcohol, furfural, benzaldehyde, 2-phenyl-2-butenal, 5-methyl-2-phenyl-2-hexenal, ethyl acetate, isobutyl acetate, ethyl l-lactate, diethyl succinate, ethyl octanoate, ethyl phenylacetate, ethyl hexadecanoate, 2-octanol (2-O, internal standard, IS), and n-Alkane mixtures (C7–C40). Solid phase microextraction (SPME) fibers, including the 50/30 μm DVB/CAR/PDMS fiber, 85 μm PA fiber, 100 μm PDMS fiber, and 7 μm PDMS fiber, were supplied by Merck (Darmstadt, Germany). Analytical-grade sodium chloride was purchased from Sigma-Aldrich Co. (Shanghai, China). Pure water was generated in the lab by the Milli-Q purification system (Millipore, Bedford, MA).

### 2.2 Samples

A total of 9 wines were analyzed in this study, falling mainly into three broad categories: ACRW (Xijiao), MCRW, and grape wine. Three samples of Xijiao with different cellar times were supplied as gifts by Shenzhen Haohao Biotechnology Co., Ltd., Shenzhen, CN: Yuanjiang (YJ, 3–5 months), Jinjiu (1 year), and Nverhong (NEH, more than 1.5 years). According to GB/T 2–1366, 2018, the physicochemical indicators of Xijiao have been measured (Fig. S1). These indicators include alcohol content, total acids, total sugar, pH value, non-sugar solidity, and total nitrogen. It was found that all measured indexes meet the criteria for Hakka rice wine, ensuring the security and quality of Xijiao samples. The MCRW samples cover three top sellers in China: Guyuelongshan (GLS, Zhejiang Guyuelongshan Shaoxing Wine Co., Ltd., Shaoxing, CN), Shikumen (SKM, Shanghai Jinfeng Wine Co., Ltd., Shanghai, CN), Kuaijishan (KJS, Kuaijishan Shaoxing Wine Co., Ltd., Shaoxing, CN). Xianhuo (XH, Shiyan Wudangjunmozui Culture Media Co., Ltd., Shiyan, CN), a kind of MCRW claiming to be ACRW also served as subjects in the study. The grape wine samples include Changcheng (CC, COFCO Wines & Spirits Co., Ltd., Beijing, CN) and Zhangyu (ZY, Yantai Changyu Co., Ltd., Yantai, CN). All samples were stored at 4 °C for analysis.

### 2.3 Sensory evaluation

The experimental protocol was referred to a previous study with minor modifications [8,19]. First, we recruited ten panelists (5 males and 5 females, between 21 and 34 years old) from SUSTech (Shenzhen, China), who met the criteria of having a good sense of smell, no rhinitis, and no alcohol allergy. Then, they underwent a two-week training period (30 min/day) to learn how to recognize and describe a series of odors. The training included 13 representative flavor description words from the aroma profiles of CRW and grape wine that had been established previously. The formal sensory evaluation took place in a closed room at approximately 20 °C. Each sample (10 mL) was placed in a sealed opaque glass bottle and randomly labeled with letters A, B, C, D, or E. Using a sensory evaluation questionnaire, the panelists were assigned samples randomly and were required to sniff them, record the aroma words, and rate the aroma intensity using a continuous scale from 0 (not perceivable) to 5 (very intense) to represent the degree of olfactory characteristics (Table S1). After the initial evaluation, each panelist was given 10 s to smell coffee beans and took a 5-min break to refresh their sense of smell. The evaluation process was repeated three times for each sample. Subsequently, the first eight aroma words with the highest frequency were selected and defined as the aroma thesaurus, including alcoholic, fruity, floral, cocoa, grain-like, sour, woody, and caramel-like. The QDA data related to the aroma thesaurus were analyzed using GraphPad Prism 9 (Dotmatics, La Jolla, CA, USA), applying One-way ANOVA and Tukey's multiple comparisons test to identify significant differences in aroma characteristics between samples. Finally, the statistical results were visualized using a radar plot created in Excel2019 (Microsoft, Seattle, WA, USA).

### 2.4 Separation and enrichment of volatile compounds

To prepare the samples, 10 mL of each sample was transferred into headspace vials (20 mL). Next, the vials were sealed using 18 mm magnetic screw craps equipped with a polytetrafluoroethylene white silicone septum. To each sample, 400 μL of the internal standard (2-octanol) was added, resulting in a final concentration of 328.8 μg/L. Additionally, 3 g of NaCl was included in the samples. The samples were thoroughly mixed to ensure that the components inside the vial were evenly distributed. Once mixed, the sample vials were placed on a custom metal bath (Jingfei Instrument Technology Co., Ltd., Hangzhou, CN) and preheated to reach equilibrium at a temperature of 50 °C for 25 min. Following equilibration, the SPME fiber was positioned 1 cm from the liquid surface of the sample vial for a duration of 30 min. Prior to this, four different SPME fibers were aged at desired temperatures according to the manufacturer's instructions. These fibers were then utilized respectively to extract the volatile substances.

### 2.5 Identification and semi-quantitation of volatile compounds by GC-MS

After extraction, the volatile substances absorbed by the SPME fiber were thermally released into the GC injection port with the splitless mode at 250 °C for 7 min holding. Agilent 8890 GC system equipped with an Agilent 5977B GC mass selective detector was used for volatiles separation and identification (Agilent, Santa Clara, CA, USA). The molecules were separated on a Zebron-5HT INFERNO capillary column (35 m × 0.25 mm × 0.1 μm) (Phenomenex, Torrance, CA, USA). The carrier gas was helium with a flow rate of 1 mL min-1. The mass spectrometer used an ionization source at 250 °C. The oven temperature was programmed to be 35 °C for the first 3 min, ramp up to 100 °C at 3 °C/min and maintained for 1 min, then to 230 °C at 8 °C/min, and finally hold for 1 min, giving a total run time of 48 min. After running, the result files were imported into UnknownAnalysis software (Agilent, Santa Clara, CA, USA) and integrated automatically. The retention index (RI) was acquired using alkane mixtures C7–C30 standard and calculated according to equation (1). Identification of unknowns was conducted by matching with NIST 2019 library (NIST, Gaithersburg, MD, USA) based on mass spectrum information and RI values. For validation purposes, interested compounds were further identified using pure standards.

(1) $$\begin{array}{c} {RI}_{x\;}=100\times Z+\frac{{RT}_{x\;}-\;{RT}_{z}}{{RT}_{z+1}-\;{RT}_{z}} \end{array}$$

where RT_z_ is the retention time of normal alkane with carbon number z, and RT_z+1_ represents the retention time of normal alkane with carbon number z + 1. The mass spectrum peak of x appears between that of normal alkane z and z + 1.

For quantitation analysis, semi-quantification of the volatile substances was performed using 2-octanol as the internal standard. The measurement was repeated three times for each analyte. The concentration of each compound was calculated using the ratio between the peak area of 2-octanol and that of the volatile compound. The equation is shown as the following (2):

(2) $$\begin{array}{c} C\left(\mu g/L\right)=\frac{A_{c}}{A_{is}}\times C_{is}\left(\mu g/L\right) \end{array}$$

where C is the concentration of each analyte (expressed as μg/L), and Cis represents the final concentration of the internal standard (2-octanol). Ac and Ais are the mass peak area of the analyte and internal standard respectively.

### 2.6 Calculation of relative odor activity values (ROAVs)

Calculation of relative odor activity values (ROAVs) is useful in characterizing the key odorants to the holistic aroma feature. It enables us to evaluate the contribution of every single volatile compound. The corresponding formula for calculating ROAVs is given as follows (3):

(3) $$\begin{array}{c} ROAV=\frac{C\;\left(\mu g/L\right)}{T\;\left(\mu g/L\right)} \end{array}$$

where C indicates the relative concentration of each volatile compound identified in samples, and T represents the odor threshold of each compound in the aqueous or ethanol medium. The reference threshold values were retrieved from the book *Compilations of Odor Threshold Values in Air, Water & other Media*.

### 2.7 Statistical analysis

Statistical analysis was conducted using GraphPad Prism 9 software (Dotmatics, La Jolla, CA, USA) in which the distribution of the data was analyzed to determine the appropriate statistical test to be applied. One-way ANOVA with Tukey's multiple comparisons test was used for multiple-group comparisons. Significant differences between two groups were noted by asterisks (*p ≤ 0.05, **p ≤ 0.01, ***p ≤ 0.001, ****p ≤ 0.0001). Heatmap was plotted by TBtools (Toolbox for Biologists; version 1.1.047, Guangzhou, CN). Unsupervised multivariate statistical analyses including the principal coordinate analysis (PCoA) and Spearman's correlation analysis were performed via the online platform OmicShare (https://www.omicshare.com/) and GraphPad Prism 9 software respectively. Supervised multivariate statistical analyses including partial least-squares discriminant analysis (PLS-DA) and orthogonal partial least-squares discriminant analysis (OPLS-DA) were performed using MetaboAnalyst 5.0 (http://www.metaboanalyst.ca/MetaboAnalyst/). Chemical structures were drawn with ChemDraw Professional 20.0 software (PerkinElmer, Waltham, MA, USA).

## 3 Results and discussion

### 3.1 Sensory evaluation of ACRW (Xijiao), MCRW, and GW

**Fig. 2:**
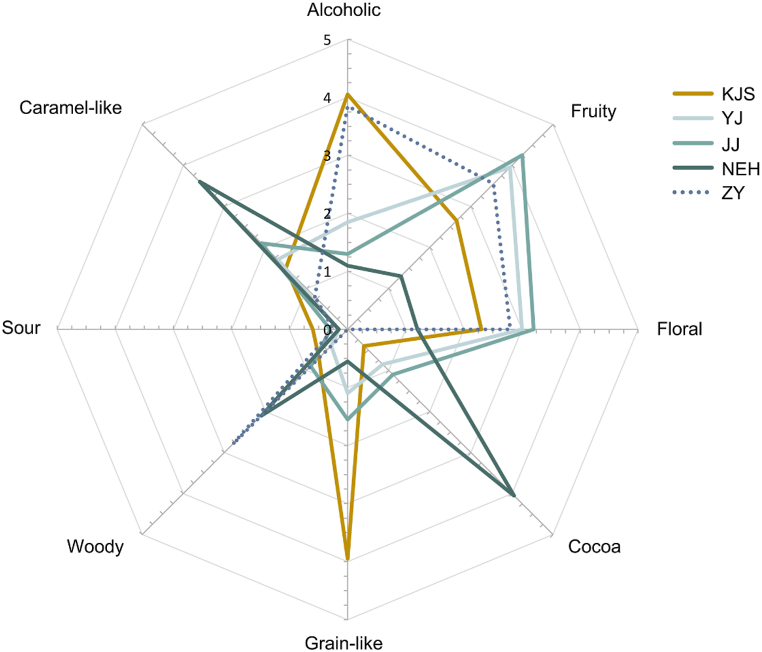
Sensory profiles of ancient Chinese rice wine (YJ, JJ, and NEH), modern Chinese rice wine (KJS), and grape wine (ZY). YJ: Yuanjiang, JJ: Jinjiu, NEH: Nverhong, KJS: Kuaijishan, ZY: Zhangyu.

To comprehensively evaluate the aroma characteristics of ACRW (Xijiao), a sensory evaluation was conducted by a trained panel of 10 assessors using quantitative descriptive analysis (QDA) (Fig. 2). To gain a better understanding of the aromatic properties of Xijiao, the MCRW and GW samples were also examined. KJS, with natural aging, was chosen to represent MCRW, whereas ZY, which undergoes biological ageing and has a similar sensory to Xijiao, was selected to represent GW. Consistent with previous studies [8,19,20], the primary aroma attributes of MCRW were described as alcoholic, grain-like, fruity, floral, and caramel-like. Specifically, KJS exhibited strong alcoholic and grain-like aromas, with slightly fruity, floral, and caramel-like aromas. In contrast, ZY had alcoholic, fruity, floral, and woody aromas. However, the aroma profile of Xijiao was more complex than that of KJS and ZY. In YJ and JJ, fruity and floral aromas were the most dominant, followed by caramel-like, grain-like, alcoholic, cocoa, woody, and sour notes. The NEH results showed a significant decrease in the intensity of fruity and floral aromas compared to YJ and JJ, while the intensity of cocoa, caramel-like, and woody fragrances notably increased, suggesting a change in flavor over time, possibly due to microbial action. When compared to KJS and ZY, YJ and JJ exhibited richer fruity and floral aromas, with a less intensity in their alcoholic and grain-like notes. Therefore, these results demonstrate that Xijiao with a short cellar time (YJ and JJ) showcases a distinctive aroma profile that combines the strong fruity and floral aromas of GW with the less intense alcoholic and grain-like aromas of MCRW. On the other hand Xijiao, with a long cellar time (NEH) is characterized by a deep cocoa- and caramel-like aroma that differs significantly from MCRW.

### 3.2 Selection of the fiber for headspace solid-phase microextraction (SPME)

To determine the volatile compounds of aromatic characteristics of each wine, HS-SPME-GC-MS analysis was conducted. The first step in this process is to select the microextraction fiber for volatile substance extraction and enrichment. We compared the extraction capacities of four commonly used fibers using YJ as a test sample. Among these fibers, the 50/30 μm DVB/CAR/PDMS fiber, a complex fiber, exhibited the best extraction capacity by extracting approximately 75 volatile compounds (Fig. S2A). The 85 μm PA fiber showed a similar extraction ability to the 100 μm PDMS fiber by identifying 50 volatile compounds. On the other hand, the 7 μm PDMS fiber showed the poorest extraction capacity, involving the lowest number of volatile compounds. Interestingly, only a small number of volatile components were detected in all the fibers, including ethanol, ethyl acetate, and phenylethyl alcohol (Fig. S2B). The 50/30 μm DVB/CAR/PDMS fiber extracted the most abundant unique volatile compounds. Additionally, only this fiber was able to cover the main aroma compounds in the CRW, such as alcohols, esters, acids, and aldehydes (Fig. S2C). Consequently, to comprehensively identify the volatile compounds, we exploited the 50/30 μm DVB/CAR/PDMS fiber for the extraction of volatiles.

### 3.3 Determination of the volatile compounds by GC-MS

**Fig. 3:**
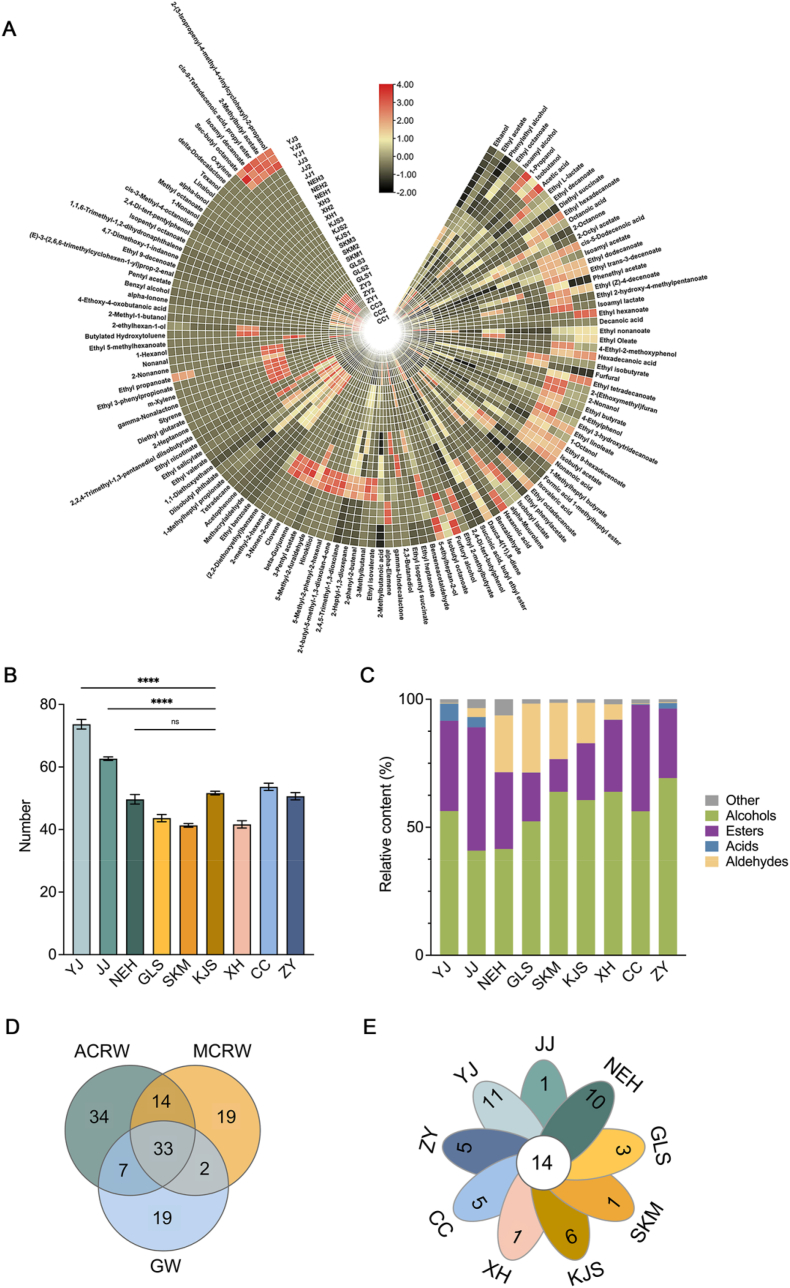
Comparison of aroma compounds identified in all samples. (A) Heatmap analysis of aroma compounds determined in all samples. Warmer colors (red) show higher concentration, while cooler colors (dark) represent lower concentration. (B) The total number of aroma compounds identified in all samples. Three independent trials were performed for each experiment. Error bars are the standard error of the mean (SEM). *p ≤ 0.05, **p ≤ 0.01, ***p ≤ 0.001, ****p ≤ 0.0001 were regarded as statistically significant. (C) The relative abundances of main aroma compounds in all samples. (D) Venn diagram of aroma compounds in ancient Chinese rice wine (Xijiao), modern Chinese rice wine (MCRW), and grape wine (GW). (E) Venn diagram of aroma compounds in all samples. YJ: Yuanjiang, JJ: Jinjiu, NEH: Nverhong, GLS: Guyuelongshan, SKM: Shikumen, KJS: Kuaijishan, XH: Xianhuo, CC: Changcheng, ZY: Zhangyu. Xijiao: Xijiao Huojiu, MCRW: modern Chinese rice wine, GW: grape wine. (For interpretation of the references to color in this figure legend, the reader is referred to the Web version of this article.)

As shown in Table 1, 128 volatile compounds were identified in all the samples. The number of aroma compounds in the MCRW samples was approximately 40, except KJS, which contained 51 compounds (Fig. 3B). The GW samples showed quantitatively similar aroma compounds, with CC containing 53 compounds and ZY containing 50 compounds. In contrast, YJ and JJ exhibited a greater variety of aromatic compounds, with 72 and 62 compounds, respectively, compared to the MCRW and GW. Importantly, the number of volatiles in NEH was lower than in YJ and JJ, suggesting weak fermentation during the ageing period. The abundance of aromatic components in YJ and JJ can be attributed to microbial activity, as shown in previous studies [4,21]. All samples shared the main types of volatile components, including esters, alcohols, acids, aldehydes, and other compounds such as ketones and terpenes. However, the proportions of these compounds varied among samples. Alcohols were the most abundant aroma compounds in almost all samples except NEH (Fig. 3C). Xijiao exhibited the most abundant specific aroma substances compared to other wines, such as isoamyl decanoate and isobutyl lactate (Fig. 3D). Additionally, each wine had unique aromatic components (Fig. 3E). Among the common aroma substances, the concentrations of 2-octanone, ethyl l-lactate, and furfural were significantly higher in Xijiao than other wines, potentially contributing to its distinct aroma features.

Alcohols, primarily synthesized through two pathways, the synthetic pathway of glucose and the Ehrlich pathway [22], are byproducts of yeast metabolism. During the cellar period, the total alcohol concentration decreased from 7844.28 μg/L to 5859.07 μg/L, showing the yeast's reduced respiratory growth and the conversion of alcohols to esters, aldehydes, and other substances. This declining trend is similar to that of MCRW with natural ageing and GW with biological aging, where alcohols decrease due to volatilization, oxidation, and esterification reactions [5,14]. We observed that ethanol, isobutanol, isoamyl alcohol, and phenylethyl alcohol were the major alcohols in YJ and JJ, providing them with alcoholic, fruity, floral, and honey-like aromas. However, NEH showed a reduction in the amount and variety of alcohols, with ethanol, isoamyl alcohol, and phenylethyl alcohol being the most common. Compared to MCRW, YJ and JJ showed a significantly wide range of alcohol types. In addition to common alcohols, YJ and JJ contained 1-propanol, 1-hexanol, 2,3-butanediol, 2-ethylhexan-1-ol, 5-ethylheptan-2-ol, 1-octanol, and 2-nonanol. Phenylethyl alcohol, a key aroma compound with a stagnant rose and honey odor in fermented beverages [10], was detected at noticeable levels in all samples. Xijiao showed the lowest phenylethyl alcohol content compared to the MCRW and GW samples (Fig. 3A). Several underlying mechanisms may contribute to these differences. Firstly, the low content of tyrosine, tryptophan, and phenylalanine in Xijiao can lead to a low concentration of phenylethyl alcohol, as they are precursors of this alcohol. Secondly, phenylethyl alcohol in alcoholic beverages is mainly produced by *Saccharomyces cerevisiae* [23]. Consequently, low yeast or enzyme activity can result in low phenylethyl alcohol content. Third, microbial activity can convert phenylethyl alcohol to other flavor compounds, such as aldehydes, which are supported by the elevated aldehyde content in NEH.

Esters, which impart fruity, floral, and sweet aromas to alcoholic beverages, were the second most dominant aroma substances in all samples [24]. These esters are primarily derived from the esterification reaction of alcohols and fatty acids or are synthesized via alcohol acetyltransferase using acetyl-CoA and higher alcohols as substrates in microbial cells [25]. The total esters determined in YJ, JJ, and NEH were 4910.12, 8092.48, and 4228.98 μg/L, respectively. The initially increasing and then decreasing trend of esters may be attributed to the vigorous microbial action at the beginning of the cellar period, which gradually diminishes over time. This trend is similar to the dynamics of esters in the GW during biological aging. Researchers have considered high hydrophobicity and thermodynamic instability as the main reasons for their decrease with ageing [26]. The release of hydrolytic enzymes, as well as the limited availability of precursors such as octanoic acid, can also contribute to a decrease in esters during biological ageing [27]. In contrast, esters first decreased and then increased during the natural ageing process of MCRW. At the beginning of aging, esters are hydrolyzed because of the instability of the new wine system. The major esters identified in YJ and JJ were ethyl octanoate, ethyl acetate, ethyl l-lactate, diethyl succinate, ethyl 2-hydroxy-4-methylpentanoate, ethyl decanoate, and isoamyl lactate. The predominant esters in NEH were ethyl acetate, diethyl succinate, ethyl decanoate, ethyl octanoate, ethyl isovalerate, and 2-octyl acetate. These results support previous findings that ethyl esters, primarily synthesized through an enzymatic transformation during yeast fermentation and ethylation of acetyl coenzyme A during fatty acid biosynthesis or degradation, are the most prevalent esters in CRW [28]. YJ and JJ contained considerably higher or similar concentrations of several esters than GW, including ethyl l-lactate, ethyl octanoate, ethyl decanoate, ethyl 2-hydroxy-4-methylpentanoate, ethyl 2-methylbutyrate, and isoamyl lactate, which contributed to the fruity and floral aroma of the fermented beverages and played a critical role in the development of the fruity and floral aroma attributes of YJ and JJ. Among all samples, the highest concentration of ethyl l-lactate, which is produced by the esterification of ethyl alcohol by lactic acid via lactic acid bacteria, was observed in JJ, indicating its major contribution to the fruity and floral aromas in YJ and JJ. This may have resulted from vibrant microbial activity or a relatively high abundance of lactic acid bacteria. Notably, ethyl hexadecanoate, a long-chain fatty acid ethyl ester, was detected at appreciable levels in Xijiao and CC and has been reported to result in waxy and creamy aroma in MCRW and GW [5].

Acetic acid, octanoic acid, *cis*-5-dodecenoic acid, decanoic acid, hexadecanoic acid, nonanoic acid, isovaleric acid, hexanoic acid, and 2-methylbutanoic acid were identified in YJ and JJ with varying levels. However, NEH only contained trace amounts of 2-methylbutanoic acid. The reduced levels of acids in NEH may be due to their involvement in esterification reactions during the cellar period. This trend mirrors the decline in acids in MCRW and GW as they age, likely due to esterification reactions of acids and alcohols [5,27]. The MCRW samples contained fewer acids compared to Xijiao, and ZY showed a lower diversity of acids than Xijiao. Acetic acid was detected at high concentration only in YJ, potentially influencing the overall sensory profile of YJ. The abundance of acetic acid in YJ could be attributed to the vigorous activity of acetic acid bacteria during the initial fermentation. JJ and NEH did not have detectable levels of acetic acid, possibly due to the formation of ethyl esters. YJ and JJ showed similar concentrations of octanoic acid as GW, contributing to fatty, cheesy, and fruity-rancid odors. Octanoic acid has been suggested to have antibacterial, antiviral, anti-algal, and anti-protozoan activities, potentially benefiting patients with cardiac diseases and neurological disorders [29].

Aldehydes, an essential component of the aroma profile of CRW, can be produced through the oxidation of alcohols or by microbial activity on amino acids. It coordinates the release of aromas and provides sweet, almond, burnt, and nutty aromas [30]. Consistent with previous studies, furfural and benzaldehyde were the predominant aldehydes in the MCRW samples [8,24,31,32]. Furfural, a key breakdown product of carbohydrates, was present in all samples and contributed to aromas reminiscent of almonds, baked potatoes, bread, burnt, and caramel. Another significant aldehyde, benzaldehyde, has been reported to have a synergistic effect with furfural because of its similar cyclic structure [10]. Low levels of aldehydes were detected in YJ and JJ samples. However, the concentrations of aldehydes in NEH were significantly higher compared to the YJ sample, increasing from 31.74 μg/L to 3131.71 μg/L. This trend mirrored that observed in MCRW and GW during the ageing process. Interestingly, the increase in aldehydes in GW during biological ageing may be attributed to a reduction reaction [26]. The abundance of furfural, benzeneacetaldehyde, 5-methyl-2-phenyl-2-hexenal, and 2-methyl-2-hexenal presumably contributed to the cocoa- and caramel-like aroma properties of NEH.

In addition to the major odorous compounds mentioned earlier, several other volatile substances, such as ketones, phenols, and terpenes, have been identified. Among these, 2-octanone was consistently detected in all samples at significant levels and exhibited fruity, floral, fatty, nutty, and cheesy aromas. Additionally, low levels of 4-ethylphenol, a volatile phenol, were detected in the Xijiao and GW samples. Previous studies have shown that 4-ethylphenol has adversely affected the aroma of grape wines by introducing horsy, barnyard, smoky, and medicinal aromas. The production of 4-ethylphenol by Dekkera/Brettanomyces yeasts occurs when they utilize free hydroxycinnamic acids and their ethyl esters as substrates [33]. Despite being considered a contaminant in GW, 4-ethylphenol may contribute to the medicinal, phenolic, and woody odors present in the CRW samples (see Table 1).

### 3.4 Identification of the key odorants by calculating relative odor activity value (ROAV)

Given the significant impact of odor threshold on odor perception, we were unable to determine the individual contributions of odorants to the overall aroma profile based solely on the concentration of each aroma compound. The relative odor activity value (ROAV) has been proposed to explain how to evaluate the contribution of individual volatile compounds to the overall aroma by the relative concentration of volatile compounds [34,35]. Generally, odorants with an ROAV greater than 1 are considered key contributors to the overall aroma profile. The results from Table 2 suggested that a total of 14, 20, and 16 volatile compounds were identified as key odorants of YJ, JJ, and NEH, respectively. Consistent with previous research [8,10,31], ethyl acetate, ethyl octanoate, and phenylethyl alcohol were identified as key odorants in the MCRW samples due to their relatively higher ROAVs. Ethyl acetate, ethyl isobutyrate, and ethyl octanoate were the predominant odorants in the GW samples. In YJ and JJ, ethyl octanoate, ethyl acetate, ethyl isobutyrate, and isoamyl lactate exhibited significantly higher ROAVs compared to other aroma components, contributing to a fruity fragrance reminiscent of pineapple, banana, and apple. Similarly, in NEH, ethyl isobutyrate, ethyl acetate, and ethyl octanoate were also identified as vital odorants. Furthermore, benzeneacetaldehyde, gamma-undecalactone, isoamyl alcohol, 5-methyl-2-furaldehyde, and 3-methylbutanal were found to contribute to the specific flavor of NEH, each imparting distinct aromas such as chocolate, nutty, grain, caramel, and malty notes. While some volatile compounds had ROAVs lower than 1, their potential effects on aroma profiles should not be overlooked. The complete ROAVs list can be found in the Supplemental Material (Table S2).

**Table 1:** Semi-quantitative results of volatile compounds identified in all ^c^amples.

Aroma c^b^mpou^d^d	CAS	Identification^a^	Concentration (±SD, μg/L)^b^									Odor description^c^
			YJ	JJ	NEH	GLS	SKM	KJS	XH	CC	ZY	
***Acids***												
Acetic acid	64-19-7	MS, Std	589.79 ± 90.09	-d	–	–	–	–	–	–	–	Pungent, sour, vinegar-like
Isovaleric acid	503-74-2	MS, RI	8.06 ± 1.47	7.02 ± 1.29	–	26.1 ± 2.19	6.22 ± 0.11	16.84 ± 0.95	12.31 ± 0.97	6.26 ± 0.57	11.83 ± 0.94	Penetrating, disagreeable, rancid, cheesy
2-Methylbutanoic acid	116-53-0	MS, RI	–	5.44 ± 0.15	6.58 ± 1	5.47 ± 1.13	4.8 ± 0.09	–	8.94 ± 0.21	5.72 ± 0.92	12 ± 0.87	Butter, cheesy, fermented, sour
Hexanoic acid	142-62-1	MS, RI	6.49 ± 0.24	–	–	–	–	–	–	–	–	Unpleasant, cheesy, sweat-like, characteristic goat-like
Octanoic acid	124-07-2	MS, RI, Std	184.89 ± 2.69	547.85 ± 21.97	–	–	–	–	–	–	477.59 ± 25.82	Unpleasant, fruity-acid
Nonanoic acid	112-05-0	MS, RI	11.32 ± 0.47	9.48 ± 0.38	–	–	–	–	–	–	–	Waxy, dirty,^e^cheesy, dairy
Decanoic acid	334-48-5	MS, RI, Std	28.73 ± 0.93	12.56 ± 1.38	–	–	–	–	–	74.99 ± 4.47	265.68 ± 30.75	Unpleasant, rancid, creamy
*cis*-5-Dodecenoic acid	2430-94-6	MS, RI	79.68 ± 4.45	69.96 ± 0.64	–	–	–	–	–	–	–	n. f^e^
Hexadecanoic acid	57-10-3	MS, RI, Std	24.41 ± 0.8	18.71 ± 0.59	–	–	–	–	–	–	–	Odorless, slight characteristic
**Total**			933.38	671.03	6.58	31.58	11.02	16.84	21.25	86.97	767.10	
***Alcohols***												
Ethanol	64-17-5	MS	4288.48 ± 236.67	3113.95 ± 240.56	2816.47 ± 254.04	7498.69 ± 239.41	8213.84 ± 320.67	11611.9 ± 465.99	6613.81 ± 246.82	9636.02 ± 334.04	10335.53 ± 393.95	Strong alcoholic
1-Propanol	71-23-8	MS, Std	611.98 ± 93.01	–	–	–	–	–	–	–	–	Mild, alcohol-like
Isobutanol	78-83-1	MS, Std	607.46 ± 97.9	149.03 ± 10.82	–	–	856.26 ± 52.95	613.5 ± 17.69	560.2 ± 36.79	–	153.02 ± 10.91	Penetrating, wine-like, disagreeable
2-Methyl-1-butanol	137-32-6	MS	–	–	–	–	–	–	–	834.47 ± 17.6	232.82 ± 26.88	Fish oil, green, malt, onion, wine
Isoamyl alcohol	123-51-3	MS, Std	1062.82 ± 104.7	779.39 ± 42.58	724.04 ± 23.08	822.04 ± 18.44	699.58 ± 43.53	1490.05 ± 154.48	1209.57 ± 114.25	1737.44 ± 167.23	3207.18 ± 133.65	Apple, brandy, spicy
1-Hexanol	111-27-3	MS, RI	–	–	–	–	–	10.28 ± 0.71	–	27.39 ± 2.23	179.28 ± 16.13	Banana, floral, grass, herb
2,3-Butanediol	513-85-9	MS, RI	2.27 ± 0.27	–	–	–	–	–	48.81 ± 5.1	–	–	Ripe fruit, buttery
2-ethylhexan-1-ol	104-76-7	MS, RI	2.59 ± 0.18	–	–	–	–	–	10.13 ± 0.15	7.05 ± 1.01	–	Mild, oily, sweet, rose
Benzyl alcohol	100-51-6	MS, RI	–	–	–	–	–	–	–	61.41 ± 6.02	76.58 ± 7.7	Boiled cherries, moss, roasted bread, rose
5-ethylheptan-2-ol	19780-40-6	MS, RI	3.49 ± 0.59	–	–	–	–	–	–	–	–	n. f
1-Octanol	111-87-5	MS, RI	18 ± 2.5	34.08 ± 1.72	–	–	–	23.18 ± 1.57	18.39 ± 1.07	10.62 ± 1.3	42.1 ± 3.99	Penetrating, rose, orange, lemon
2-Nonanol	628-99-9	MS, RI	19.31 ± 2.55	81.9 ± 1.87	–	–	–	–	17.03 ± 1.14	–	11.78 ± 0.55	Cucumber, green, fatty, melon
Phenylethyl alcohol	60-12-8	MS, RI, Std	1227.89 ± 179.41	2701.61 ± 166.29	2318.56 ± 196.56	6784.59 ± 253.74	6806.08 ± 323.16	9160.32 ± 217.63	6211.67 ± 155.8	4404.08 ± 353.92	9982.53 ± 151.99	Rose, honey
1-Nonanol	143-08-8	MS, RI	–	–	–	–	–	–	–	13.42 ± 1.42	59.03 ± 6.3	Fat, floral, green, oil
Texanol	77-68-9	MS, RI	–	–	–	–	–	–	–	–	10.68 ± 0.7	Mild, characteristic
**Total**			7844.28	6859.95	5859.07	15105.31	16575.76	22909.24	14689.63	16731.91	24290.54	
***Aldehydes***												
Methacrylaldehyde	78-85-3	MS	–	–	–	194.33 ± 6.26	206.12 ± 7.26	141.03 ± 6.89	–	–	–	Pungent, characteristic
3-Methylbutanal	590-86-3	MS	–	–	297.7 ± 29.06	–	–	–	–	–	–	Apple, peach
1,1-Diethoxyethane	105-57-7	MS	–	–	–	20.4 ± 1.6	20.74 ± 0.86	20.59 ± 0.86	9.16 ± 0.67	–	–	Creamy, fruit, pleasant, tropical fruit
Furfural	98-01-1	MS, RI, Std	22.75 ± 2	481.31 ± 30.57	2165.72 ± 119.42	1639.8 ± 116.08	1556.05 ± 181.79	1672.75 ± 152.64	1076.34 ± 43.99	27.54 ± 2.49	67.89 ± 7.52	Almond, baked potatoes, bread, burnt, caramel
2-methyl-2-hexenal	28467-88-1	MS, RI	–	–	6.46 ± 0.31	–	–	–	–	–	–	n. f
Benzaldehyde	100-52-7	MS, RI, Std	6.33 ± 0.8	61.09 ± 2.24	86.29 ± 2.35	5757.2 ± 212.73	3824.61 ± 259.36	3875.68 ± 243.83	244.66 ± 37.4	–	–	Sweet, cherry, almond, nutty
5-Methyl-2-furaldehyde	620-02-0	MS, RI	–	–	40.29 ± 5.39	–	–	–	–	–	–	Spicy-sweet, warm, caramel
Benzeneacetaldehyde	104-76-7	MS, RI	2.66 ± 0.1	50.52 ± 5.26	192.13 ± 9.01	–	–	46.73 ± 1.82	57.32 ± 6.78	–	–	Floral, sweet, hyacinth
Nonanal	124-19-6	MS, RI	–	–	–	–	–	26.36 ± 1.04	–	–	–	Fat, floral, green, lemon
2-phenyl-2-butenal	4411-89-6	MS, RI, Std	–	–	236.8 ± 18.94	150.5 ± 6.45	101.94 ± 8.11	172.67 ± 9.65	–	–	–	Cocoa, roasted, rum, sweet
(E)-3-(2,6,6-trimethylcyclohexen-1-yl)prop-2-enal	4951-40-0	MS, RI	–	–	–	–	–	–	–	41.04 ± 3.83	42.65 ± 1.94	n. f
5-Methyl-2-phenyl-2-hexenal	21834-92-4	MS, RI, Std	–	–	77.95 ± 1.58	–	–	–	–	–	–	Cocoa, roasted, sweet
**Total**			31.74	592.92	3131.71	7762.23	5709.46	5954.80	1387.48	68.58	110.53	
***Esters***												
Ethyl acetate	141-78-6	MS, Std	1406.99 ± 267.78	1694.56 ± 103.31	1893.9 ± 192.73	1786.61 ± 24.6	883.01 ± 33.12	1770.42 ± 158.6	1868.87 ± 135.24	4691.38 ± 260.42	1265.23 ± 58.71	Fruity, pineapple, apple, banana
Ethyl propanoate	105-37-3	MS	34.43 ± 1.02	5.43 ± 0.21	–	–	–	29.46 ± 1.42	8.9 ± 0.51	–	–	Apple, pineapple, rum, strawberry
Ethyl isobutyrate	97-62-1	MS	23.17 ± 1.68	21.92 ± 1.47	91.72 ± 2.91	–	–	–	15.79 ± 2.11	56.94 ± 2.45	–	Fruity, pleasant
Isobutyl acetate	110-19-0	MS, Std	16.54 ± 0.83	16.1 ± 0.81	7.48 ± 0.63	–	–	–	11.74 ± 0.45	–	–	Fruity, currant, pear, floral, hyacinth, rose
Ethyl butyrate	105-54-4	MS	18.85 ± 0.37	36.37 ± 2.11	21.33 ± 1.08	27.51 ± 1.44	–	14.98 ± 0.12	21.95 ± 1.34	6.69 ± 1.52	9.7 ± 0.53	Fruity, pineapple, apple, banana
Ethyl l-lactate	687-47-8	MS, Std	483.67 ± 33.47	1275.6 ± 161.89	33.71 ± 2.73	352.18 ± 38.3	154.5 ± 10.22	684.09 ± 20.29	522.79 ± 50.7	70.38 ± 8.64	343.92 ± 31.51	Rum, fruity, creamy, fatty
Ethyl isovalerate	108-64-5	MS, RI	–	12.86 ± 0.86	118.14 ± 13.32	44.03 ± 2.19	15.68 ± 0.45	–	–	6.2 ± 0.88	6.75 ± 0.67	Fruity, vinous, apple
Ethyl 2-methylbutyrate	7452-79-1	MS, RI	4.43 ± 1.14	25.09 ± 2.86	–	–	–	–	–	7.64 ± 0.36	7.47 ± 0.27	Fruity, green apple, kiwi, strawberry
3-Pentyl acetate	620-11-1	MS, RI	–	–	36.21 ± 5.94	–	12.98 ± 0.61	–	49.69 ± 4.56	–	–	n. f
Pentyl acetate	628-63-7	MS, RI	–	–	–	–	–	–	–	46.93 ± 3.27	–	Banana
Isoamyl acetate	123-92-2	MS, RI	71.66 ± 5.22	146.44 ± 0.88	83.4 ± 1.73	34.79 ± 1.53	18.51 ± 0.65	17.73 ± 0.75	109.16 ± 15.1	94.3 ± 6.96	90.75 ± 8.99	Fruity, pear, banana-like, sweet, fragrant
2-Methylbutyl acetate	624-41-9	MS, RI	25.34 ± 1.19	–	–	–	–	–	–	–	–	Apple, banana, pear
Ethyl valerate	539-82-2	MS, RI	–	–	–	17.92 ± 0.98	–	17.63 ± 0.77	–	–	–	Apple, dry fish, herb, nut, yeast
Isobutyl lactate	585-24-0	MS, RI	7.38 ± 0.8	24.81 ± 0.31	–	–	–	–	–	–	–	Buttery, caramel, fruity
Ethyl hexanoate	123-66-0	MS, RI	30.56 ± 1.28	–	–	–	–	–	–	–	–	Fruity, apple peel, brandy, fruit gum, overripe fruit, pineapple
Ethyl 2-hydroxy-4-methylpentanoate	10348-47-7	MS, RI	40.45 ± 1.33	409.27 ± 27.37	–	94.49 ± 6.53	55.26 ± 2.84	371.95 ± 20.47	81.54 ± 8.97	12.68 ± 1.34	30.42 ± 1.93	Fruity, blue berry, tropical fruit, lime, valerian oil
Isoamyl lactate	19329-89-6	MS, RI	35.94 ± 1.97	400.42 ± 9.94	–	32.23 ± 3.65	16.95 ± 0.34	65.55 ± 5.76	50.76 ± 3.1	16.18 ± 2.02	86.9 ± 3.98	Fruity
Formic acid 1-methylheptyl ester	1000368-94-5	MS, RI	8.87 ± 1.5	–	18.98 ± 1.67	28.49 ± 1.05	23.78 ± 0.73	40.78 ± 2.3	44.41 ± 1.84	–	–	n. f
Ethyl 5-methylhexanoate	10236-10-9	MS, RI	–	–	–	–	4.43 ± 0.47	–	–	–	–	Fruity, caramel
Ethyl heptanoate	106-30-9	MS, RI	2.43 ± 0.24	8.6 ± 1.12	3.26 ± 1.05	41.82 ± 2.83	7.65 ± 0.66	40.58 ± 1.59	9.93 ± 0.64	–	–	Pineapple, brandy
Methyl octanoate	111-11-5	MS, RI	–	–	–	–	–	–	–	3.66 ± 0.47	–	Fruit, orange, wax, wine
2-Octyl acetate	2051-50-5	MS, RI	82.54 ± 5.09	109.64 ± 7.91	115.21 ± 5.93	164.06 ± 7.81	121.57 ± 15.94	162.18 ± 11.93	178 ± 28.17	60.61 ± 4.26	19.2 ± 1.38	Fruity
Ethyl benzoate	93-89-0	MS, RI	–	–	–	573.06 ± 22.84	403.33 ± 32.17	450.7 ± 24.96	–	–	–	Camomile, celery, fat, flower, fruit
Diethyl succinate	123-25-1	MS, RI, Std	385.27 ± 13.03	924.27 ± 96.63	887.32 ± 18.15	1476.94 ± 96.79	1030.72 ± 96.08	3288.37 ± 235.31	1823.67 ± 110.46	2553.84 ± 182.5	5271.51 ± 183.58	Pleasant, floral, fruity, wine
Ethyl octanoate	106-32-1	MS, RI, Std	1159.84 ± 136.38	1720.39 ± 92.13	332.63 ± 33.02	270.74 ± 18.52	181.02 ± 27.59	316.83 ± 15.39	1192.13 ± 162.3	2784.79 ± 196.1	1276.1 ± 93.94	Winey, brandy, fruity, pineapple, apricot
Ethyl nicotinate	614-18-6	MS, RI	–	–	–	15.49 ± 1.09	8.03 ± 0.76	43.54 ± 4.64	–	–	–	Somky
Ethyl hydrogen succinate	1070-34-4	MS, RI	–	–	–	–	–	–	–	333.38 ± 19.45	–	Fruity
1-Methylheptyl propionate	1000164-41-5	MS, RI	10.19 ± 1.46	12.85 ± 0.89	–	25.91 ± 2.73	25.98 ± 0.73	25.8 ± 1.39	28.11 ± 0.8	–	–	n. f
Ethyl phenylacetate	101-97-3	MS, RI, Std	7.42 ± 1	44.44 ± 0.79	53.65 ± 1.4	285.6 ± 11.65	178.94 ± 27.43	298.52 ± 17.09	33.6 ± 1.67	18.97 ± 1.93	27.75 ± 2.32	Sweet, pleasant, honey
Phenethyl acetate	103-45-7	MS, RI	47.29 ± 5	167.81 ± 15.75	84.48 ± 9.13	65.74 ± 4.5	73.32 ± 3.7	146.65 ± 7.95	206.54 ± 34.92	94.39 ± 6.43	175.48 ± 12.8	Very sweet, rosy, honey
Ethyl salicylate	118-61-6	MS, RI	–	–	–	16.33 ± 1.3	–	–	–	–	–	Spicy, anisic, wintergreen
Diethyl glutarate	818-38-2	MS, RI	–	–	–	–	–	149.78 ± 11.71	–	–	18.67 ± 1.38	n. f
delta-Dodecalactone	713-95-1	MS, RI	–	–	–	–	–	–	–	–	10.54 ± 0.45	Fruity, peach, pear, plum
*cis*-3-Methyl-4-octanolide	55013-32-6	MS, RI	–	–	–	–	–	–	–	14.91 ± 1.99	12.1 ± 0.82	Woody
Ethyl nonanoate	123-29-5	MS, RI	28.03 ± 0.33	22.61 ± 0.86	–	35.21 ± 3.51	–	24.43 ± 0.58	–	10.65 ± 0.28	–	Fruity, grape, flroal, rose, brandy
1-Methylheptyl butyrate	20286-44-6	MS, RI	10.06 ± 2.39	9.1 ± 0.21	–	24.6 ± 2.35	22.83 ± 1.61	29.48 ± 1.06	51.71 ± 6.46	19.29 ± 2.43	–	n. f
Succinic acid, butyl ethyl ester	1000324-85-1	MS, RI	5.7 ± 0.55	8.1 ± 0.59	35.2 ± 1.97	–	–	30.56 ± 1.67	30.96 ± 1.11	21.67 ± 1.7	46.8 ± 5.09	n. f
Ethyl 3-phenylpropionate	2021-28-5	MS, RI	–	–	–	–	–	43.57 ± 2.19	–	–	–	Flower, honey
Isobutyl octanoate	5461-06-3	MS, RI	3.99 ± 0.78	9.9 ± 2	–	–	–	–	–	–	–	Fruity
*Sec*-butyl octanoate	5458-61-7	MS, RI	3.65 ± 0.79	–	–	–	–	–	–	–	–	n. f
gamma-Nonalactone	104-61-0	MS, RI	–	–	–	–	–	81.01 ± 3.08	–	–	–	Coconut, creamy, waxy, fatty, milky
gamma-Undecalactone	104-67-6	MS, RI	2.12 ± 0.57	5.78 ± 0.61	12.29 ± 0.77	–	–	60.3 ± 3.49	–	–	–	Fruity, apricot, peach
Ethyl (Z)-4-decenoate	7367-84-2	MS, RI	41.22 ± 3.01	58.82 ± 5.36	–	–	–	–	–	–	–	Fruity, pineapple, pear
Ethyl 9-decenoate	67233-91-4	MS, RI	–	–	–	–	–	–	–	28.86 ± 3.17	30.79 ± 0.47	Fruity, fatty
Ethyl decanoate	110-38-3	MS, RI	455.82 ± 16.57	403.77 ± 13.3	278.3 ± 17.77	–	9.87 ± 0.11	53.76 ± 7.79	79.37 ± 2.82	1043.64 ± 157.08	362.45 ± 25.34	Brandy, grape, pear, coconut, rose
Ethyl isopentyl succinate	28024-16-0	MS, RI	2.43 ± 0.28	63.57 ± 1.12	62.41 ± 1.4	28.63 ± 0.92	21.5 ± 0.8	62.59 ± 3.45	29.93 ± 1.88	200.63 ± 33.7	405.05 ± 30.43	n. f
Ethyl *trans*-2-decenoate	7367-88-6	MS, RI	68.14 ± 0.51	83.94 ± 1.24	–	–	–	–	–	–	–	Fatty, waxy, over-ripe pear
Isopentyl octanoate	2035-99-6	MS, RI	–	–	–	–	–	–	–	20.07 ± 0.95	–	Fruity
Ethyl 3-hydroxytridecanoate	107141-15-1	MS, RI	18.53 ± 0.81	84.24 ± 4.32	–	–	–	–	–	–	–	n. f
2,2,4-Trimethyl-1,3-pentanediol diisobutyrate	6846-50-0	MS, RI	–	–	–	14.47 ± 1.08	33.09 ± 1.1	–	–	–	–	Musty
Ethyl dodecanoate	106-33-2	MS, RI	68.64 ± 0.5	68 ± 5.54	41.71 ± 1.29	–	–	13.9 ± 0.88	–	19.23 ± 3.88	19.11 ± 0.77	fatty, fruity, floral
Isoamyl decanoate	2306-91-4	MS, RI	6.75 ± 0.85	–	–	–	–	–	–	–	–	Waxy, banana, fruity, sweet, green
*cis*-9-Tetradecenoic acid, propyl ester	1000405-14-2	MS, RI	9.29 ± 0.94	–	–	–	–	–	–	–	–	n. f
Ethyl tetradecanoate	124-06-1	MS, RI	21.75 ± 0.38	12.81 ± 0.99	–	–	–	–	–	–	–	Waxy, orris
Diisobutyl phthalate	84-69-5	MS, RI	–	–	–	25.17 ± 1.01	–	–	–	–	–	Ester-like
Ethyl 9-hexadecenoate	54546-22-4	MS, RI	17.04 ± 1.13	16.92 ± 0.86	–	–	–	–	–	–	–	Oil
Ethyl hexadecanoate	628-97-7	MS, RI, Std	190.32 ± 29.67	148.25 ± 29.57	17.65 ± 1.86	–	–	–	–	105.56 ± 10.52	–	Mild waxy, creamy
Ethyl linoleate	544-35-4	MS, RI	18.2 ± 0.9	18.49 ± 1.24	–	–	–	–	–	–	–	Mild floral, oil
Ethyl Oleate	111-62-6	MS, RI	27.57 ± 0.98	12.38 ± 1.76	–	–	–	44.4 ± 1.26	–	2.81 ± 0.42	–	Floral
Ethyl octadecanoate	111-61-5	MS, RI	7.6 ± 1.24	8.94 ± 0.31	–	–	–	–	–	3.87 ± 0.55	–	Little odor
**Total**			4910.12	8092.48	4228.98	5482.05	3302.94	8379.61	6449.53	12350.18	9516.72	
***Other***												
2,4,5-Trimethyl-1,3-dioxolane	3299-32-9	MS	–	–	103.43 ± 10.29	54.04 ± 3.77	34.37 ± 1.97	74.04 ± 4.51	62.59 ± 5.09	–	–	Phenolic, astringent, drying
Furfuryl alcohol	98-00-0	MS, RI	4.33 ± 0.92	–	–	–	–	–	–	–	–	Burnt, caramel, cooked
2-Heptyl-1,3-dioxepane	61732-92-1	MS, RI	–	–	188.74 ± 14.95	114.84 ± 5.08	59.98 ± 1.46	–	110.22 ± 15.61	–	–	n. f
2-*t*-butyl-5-methyl-1,3-dioxolan-4-one	130930-47-1	MS, RI	–	–	77.97 ± 9.26	–	–	–	–	–	–	n. f
(2,2-Diethoxyethyl)benzene	6314-97-2	MS, RI	–	–	3.75 ± 0.77	12.02 ± 0.51	–	–	–	–	–	Fragrant
Tetradecane	629-59-4	MS, RI	–	–	–	43.87 ± 3.33	28.66 ± 1.5	–	–	–	–	Fragrant
m-Xylene	108-38-3	MS, RI	–	–	–	–	–	77.11 ± 4.76	–	–	–	Sweet, aromatic
Styrene	100-42-5	MS, RI	–	–	–	–	–	116.28 ± 9.3	–	–	–	Sweet, floral, balsamic
O-xylene	95-47-6	MS, RI	–	–	–	–	–	–	–	–	8.13 ± 0.4	Sweet, aromatic
2-(Ethoxymethyl)furan	1000450-02-5	MS, RI	21.01 ± 0.56	100.99 ± 3.68	12.06 ± 2	–	16.63 ± 0.8	–	–	6.35 ± 0.39	17.44 ± 1.13	Sweet, nutty, spicy
1,1,6-Trimethyl-1,2-dihydronaphthalene	30364-38-6	MS, RI	–	–	–	–	–	–	–	23.08 ± 2.86	21.53 ± 1.09	Fragrant
4-Ethylphenol	123-07-9	MS, RI	18.82 ± 2.53	89.45 ± 5.56	24.94 ± 3.09	–	–	–	–	99.7 ± 6.72	46.39 ± 2.27	Woody, phenolic, medicinal
4-Ethyl-2-methoxyphenol	2785-89-9	MS, RI	25.35 ± 3.17	86.11 ± 0.62	–	–	–	–	–	52.98 ± 4.49	–	Sweet, spicy, medicinal, clove
Butylated Hydroxytoluene	128-37-0	MS, RI	–	–	–	–	–	–	14.66 ± 0.39	–	–	Musty, cresylic-like, toasted cereal
2,4-Di-*tert*-butylphenol	96-76-4	MS, RI	5.26 ± 0.47	5.43 ± 0.79	5.36 ± 0.9	20.58 ± 2.36	17.86 ± 0.41	20.63 ± 0.83	–	22.31 ± 1.98	44.99 ± 4.68	Characteristic, alkyl phenol-like
2,4-Di-*tert*-pentylphenol	120-95-6	MS, RI	–	–	–	–	–	–	–	16.65 ± 1.75	–	n. f
Hinokitiol	55669-91-5	MS, RI	–	–	42.19 ± 4.82	–	–	–	–	–	–	Evergreen
alpha-Muurolene	10208-80-7	MS, RI	6.68 ± 0.82	15.76 ± 5.09	22.85 ± 1.22	–	–	–	–	–	–	Woody, pine, citrus
Clovene	117066-77-0	MS, RI	–	–	12.97 ± 1.9	–	–	–	–	–	–	Clove
Dauca-4(11),8-diene	395070-76-5	MS, RI	5.97 ± 0.75	6.03 ± 0.73	6.33 ± 1.24	–	–	–	–	–	–	n. f
beta-Gurjunene	17334-55-3	MS, RI	–	–	29.07 ± 6.45	–	–	–	–	–	–	Woody
alpha-Elemene	5951-67-7	MS, RI	–	18.33 ± 1.01	–	–	–	–	–	–	–	Spicy, fennel-like
2-Heptanone	110-43-0	MS, RI	–	–	–	10.28 ± 0.17	–	–	–	–	–	Blue cheese, fruit, green, nut, spice
2-Octanone	111-13-7	MS, RI	103.83 ± 5.34	254.45 ± 32.36	347.66 ± 38.44	182.59 ± 22.08	185.69 ± 17.58	180.31 ± 28.11	264.34 ± 46.21	89.19 ± 4.52	57.3 ± 5.01	Fruity, apple, floral, nutty, fatty, cheesy
Acetophenone	98-86-2	MS, RI	–	–	–	56.12 ± 3.89	16.61 ± 1.2	30.48 ± 1.31	–	–	–	Almonds, flower, meat, must
2-Nonanone	821-55-6	MS, RI	–	–	–	–	–	27.43 ± 1.51	–	–	–	Fragrant, fruit, green, hot milk
3-Nonen-2-one	14309-57-0	MS, RI	–	–	10.98 ± 1.31	–	–	–	–	–	–	Fruity, berry, wet
4,7-Dimethoxy-1-indanone	52428-09-8	MS, RI	–	–	–	–	–	–	–	28.83 ± 3.2	29.95 ± 1.09	n. f
Linalool	78-70-6	MS, RI	–	–	–	–	–	–	–	–	12.94 ± 0.85	Coriander, floral, lavender, lemon, rose
alpha-Ionone	8013-90-9	MS, RI	–	–	–	–	–	–	–	162.32 ± 7.95	101.46 ± 10.68	Sweet, floral,^a^violets, woody
alpha-Ionol	25312-34-9	MS, RI	–	–	–	–	–	–	–	–	64.62 ± 4.05	Floral, ionones
2-(3-Isop^c^openyl-4-methyl-4-vinylcyclohexyl)-2-propanol	639-99-6	MS, RI	14.04 ± 1.38	–	–	–	–	–	–	–	–	n. f
**Total**			205.30	576.55	888.30	49^e^.34	359.82	526.29	451^d^82	501.40	404.75	

a Identification of volatile compounds based on MS (mass spectrum), RI (^a^etention index), and Std (pure standard).

b Three parallel tests were performed to determine the concentrat^b^on of each aroma compound.

c Odor description words were obtained from the websites JECFA (https://www.fao.org/food-safety/scientific-advice/jecfa/en/) and The Good Scents Company (http://www.thegoodscentscompany.com/index.html).

d “-” represents “not detected”.

e “n. f” represents “not found”.

**Table 2:** Relative odor activity values (ROAVs) of key aroma compounds in all samples.

Aroma compound	Threshold (μg/L)^a^	Odor activity value (OAV)									Odor description
		YJ	JJ	NEH	GLS	SKM	KJS	XH	CC	ZY	
Octanoic acid	500.0	<1	1.10	-b	–	–	–	–	–	<1	Unpleasant, fruity-acid
Decanoic acid	130.0	<1	<1	–	–	–	–	–	<1	2.04	Unpleasant, rancid, creamy, dust, fat, grass
Ethanol	10000.0	<1	<1	<1	<1	<1	1.16	<1	<1	1.03	Strong alcoholic
Phenylethyl alcohol	250.0	4.91	10.81	9.27	27.14	27.22	36.64	24.85	17.62	39.93	Rose, honey
Isoamyl alcohol	250.0	4.25	3.12	2.90	3.29	2.80	5.96	4.84	6.95	12.83	Apple, brandy, spicy, bread, grain
2-Nonanol	58.0	<1	1.41	–	–	–	–	<1	–	<1	Cucumber, green, fatty, melon
1-Nonanol	34.0	–	–	–	–	–	–	–	<1	1.74	Fat, floral, green, oil
Benzaldehyde	350.0	<1	<1	<1	16.45	10.93	11.07	<1	–	–	Sweet, cherry, fruity, roasted, caramel, almond, nutty
Benzeneacetaldehyde	4.0	<1	12.63	48.03	–	–	11.68	14.33	–	–	Floral, sweet, hyacinth, chocolate
3-Methylbutanal	120.0	–	–	2.48	–	–	–	–	–	–	Apple, peach, malty
5-Methyl-2-furaldehyde	16.0	–	–	2.52	–	–	–	–	–	–	Spicy-sweet, warm, caramel, nutty
Methacrylaldehyde	25.0	–	–	–	7.77	8.24	5.64	–	–	–	Pungent, characteristic
Ethyl acetate	5.0	281.40	338.91	378.78	357.32	176.60	354.08	373.77	938.28	253.05	Fruity, pineapple, apple, banana
Ethyl octanoate	5.0	231.97	344.08	66.53	54.15	36.20	63.37	238.43	556.96	255.22	Fruity, brandy, winey, pineapple, apricot
Ethyl decanoate	200.0	2.28	2.02	1.39	–	<1	<1	<1	5.22	1.81	Brandy, grape, pear, coconut, rose
Diethyl succinate	1200.0	<1	<1	<1	1.23	<1	2.74	1.52	2.13	4.39	Pleasant, floral, fruity, wine
2-Octyl acetate	38.0	2.17	2.89	3.03	4.32	3.20	4.27	4.68	1.60	<1	Fruity
Isoamyl acetate	30.0	2.39	4.88	2.78	1.16	<1	<1	3.64	3.14	3.03	Fruity, pear, banana-like, sweet, fragrant
Isoamyl lactate	3.0	11.98	133.47	–	10.74	5.65	21.85	16.92	5.39	28.97	Fruity
Ethyl hexanoate	14.0	2.18	–	–	–	–	–	–	–	–	Fruity, apple peel, brandy, fruit gum, overripe fruit, pineapple
Ethyl isobutyrate	5.6	4.138	3.914	16.379	–	–	–	2.820	10.168	–	Fruity, pleasant
Ethyl butyrate	20.0	<1	1.82	1.07	1.38	–	<1	1.10	<1	<1	Fruity, pineapple, apple, banana
Ethyl phenylacetate	73.0	<1	<1	<1	3.91	2.45	4.09	<1	<1	<1	Sweet, pleasant, honey
Ethyl 2-methylbutyrate	18.0	<1	1.39	–	–	–	–	–	<1	<1	Fruity, green apple, kiwi, strawberry
Ethyl heptanoate	2.0	1.22	4.30	1.63	20.91	3.83	20.29	4.97	–	–	Pineapple, berry, plum, brandy
gamma-Undecalactone	4.0	<1	1.45	3.07	–	–	15.08	–	–	–	Fruity, apricot, peach, nutty, vanilla
Ethyl isovalerate	3.0	–	4.29	39.38	14.68	5.23	–	–	2.07	2.25	Fruity, vinous, apple
Ethyl benzoate	500.0	–	–	–	1.15	<1	<1	–	–	–	Camomile, celery, fat, flower, fruit
Ethyl valerate	5.0	–	–	–	3.58	–	3.53	–	–	–	Apple, dry fish, herb, nut, yeast
gamma-Nonalactone	65.0	–	–	–	–	–	1.25	–	–	–	Coconut, creamy, waxy, fatty, milky
2-Methylbutyl acetate	11.0	2.30	–	–	–	–	–	–	–	–	Apple, banana, pear
Styrene	100.0	–	–	–	–	-	1.16	–	–	–	Sweet, floral, balsamic
2-(Ethoxymethyl)furan	11.0	1.91	9.18	1.10	–	1.51	–	–	<1	1.59	Sweet, nutty, spicy
1,1,6-Trimethyl-1,2-dihydronaphthalene	2.5	–	–	–	–	–	–	–	9.23	8.61	Licorice, burned, tobacco, herb, petrol
4-Ethyl-2-methoxyphenol	6.9	3.67	12.48	–	–	–	–	–	7.68	–	Sweet, spicy, medicinal, clove
4-Ethylphenol	51.0	<1	1.75	<1	–	–	–	–	1.95	<1	Woody, phenolic, medicinal, sweet
alpha-Ionone	8.0	–	–	–	–	–	–	–	20.29	12.68	Sweet, floral, violets, woody
Linalool	6.0	–	–	–	–	–	–	–	–	2.16	Coriander, floral, lavender, lemon, rose

a Odor threshold values were obtained from the book Compilations of Odor Threshold Values in Air, Water & other Media.

b “-” represents “not detected”.

### 3.5 Determination of discriminant compounds by multivariate statistical analysis

**Fig. 4:**
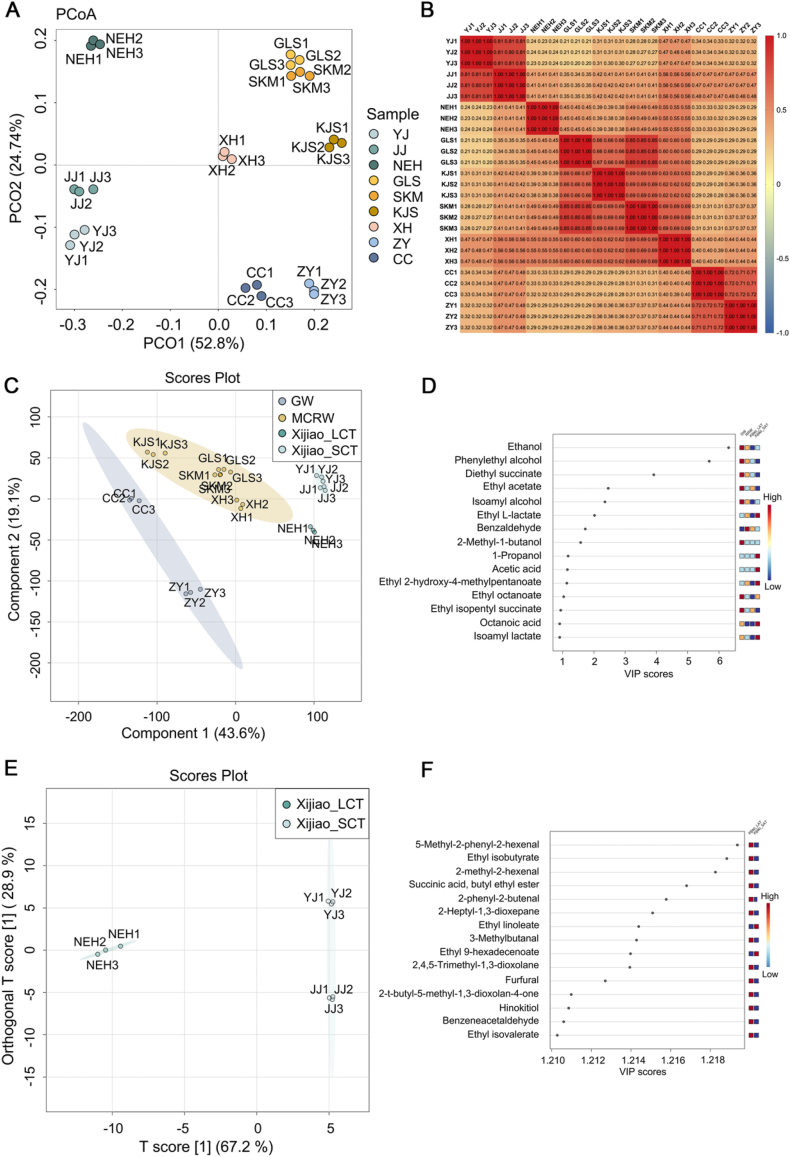
Multivariate statistical analysis of all samples based on the aroma compounds. A-B are the results of unsupervised multivariate statistical analyses. C–F are the results of supervised multivariate statistical analyses. (A) Principal coordinate analysis (PCoA) of all samples. (B) Spearman's correlation analysis of all samples. The similarity between samples is indicated by the color change and the grid value. The redder the color, the more similar the sample. When the value is closer to 1, the sample is more similar. (C) Score plot of partial least squares-discriminant analysis (PLS-DA). (D) Variable importance in projection (VIP) scores plot by PLS-DA and heatmap analysis of key odorants identified in GW, MCRW, Xijiao_LCT, Xijiao_SCT (right panel). The raw data underwent normalization using “Pareto scaling” method. “GLS1” was designated as the “reference sample” for sample normalization to enhance the interpretability of principal components within the data set. Warmer colors (red) show higher concentration, while cooler colors (blue) represent lower concentration. (E) Score plot of orthogonal partial least-squares discriminant analysis (OPLS-DA). (F) VIP scores plot by OPLS-DA and heatmap analysis of cellar markers determined in Xijiao_SCT and Xijiao_LCT (right panel). YJ: Yuanjiang, JJ: Jinjiu, NEH: Nverhong, GLS: Guyuelongshan, SKM: Shikumen, KJS: Kuaijishan, XH: Xianhuo, CC: Changcheng, ZY: Zhangyu. Xijiao_LCT: Xijiao Huojiu with long cellar time, Xijiao_SCT: Xijiao Huojiu with short cellar time, MCRW: modern Chinese rice wine, GW: grape wine. (For interpretation of the references to color in this figure legend, the reader is referred to the Web version of this article.)

Multivariate statistical analyses, incorporating both unsupervised and supervised methods, were conducted to identify the key volatile compounds responsible for the aroma distinctions among the ACRW, MCRW, and GW samples. Principal coordinate analysis (PCoA), an unsupervised method based on dimensionality reduction, was employed to visualize and analyze the semi-quantitative results of all the volatile compounds. The PCoA score plot revealed distinct regional distribution characteristics among all samples, highlighting significant aroma divergence (Fig. 4A). Particularly, YJ samples were located close to JJ samples, confirming their similar sensory attributes (Fig. 2). While the PCoA successfully segregated the samples into four groups, namely Xijiao with a short cellar time (Xijiao_SCT, YJ, and JJ), Xijiao with a long cellar time (Xijiao_LCT, NEH), MCRW, and GW, however, it did not demonstrate similarities between the groups (Fig. 4A). Spearman's correlation analysis, a statistical tool for evaluating data relationships, was employed. The analysis revealed similarities between the Xijiao_SCT (YJ and JJ), MCRW (GLS, KJS, SKM, and XH), and GW (ZY and CC) samples, whereas the NEH samples exhibited a distinct aroma profile (Fig. 4B). These results support the categorization of the samples into four groups: Xijiao_SCT, Xijiao_LCT, MCRW, and GW.

To identify the discriminant substances contributing to the aroma differences among the samples based on the new distribution of all samples according to PCoA and Spearman's correlation analysis, we adopted partial least-squares discriminant analysis (PLS-DA) to analyze the semi-quantitative data of all the aroma compounds, a supervised statistical method. Consistent with the results of the unsupervised analysis, the samples were distributed across the four regions, suggesting an observable difference in the aroma composition (Fig. 4C). However, the disadvantage of such a supervised analysis is the risk of overfitting. To ensure the accuracy and suitability of the model, we performed k-fold cross-validation and permutation tests. Cross-validation, also called rotation estimation or out-of-sample testing, is a similar model validation technique for assessing how the results of a statistical analysis can be generalized to an independent dataset [36]. The R^2^ and Q^2^ values, used to evaluate the model's applicability and predictability, were both above 0.5 and close to 1, reflecting the reliability of the PLS-DA model (Fig. S3A). A permutation test was then conducted to validate the model. The p-value suggested that the probability of obtaining a better model was less than 0.01 in 100 permutation tests (Fig. S3B). Overall, the results of the k-fold cross-validation and permutation tests confirmed the reliability of the PLS-DA model, ruling out any overfitting issues and demonstrating its ability to effectively discriminate among all samples.

The variable injection value (VIP) was used to characterize the most dominant variables. VIP score is a weighted sum of squares of the PLS loadings. The weights are based on the amount of explained Y-variance in each dimension. Variables with a VIP greater than 1 were considered having a significant effect on the discrimination of all samples [37]. The results of the VIP score plot revealed that a collection of 12 volatile compounds could distinguish Xijiao_SCT, Xijiao_LCT, MCRW, and GW well (Fig. 4D). These included ethanol, phenylethyl alcohol, diethyl succinate, ethyl acetate, isoamyl alcohol, ethyl l-lactate, benzaldehyde, 2-methyl-1-butanol, 1-propanol, acetic acid, ethyl 2-hydroxy-4-methylpentanoate, and ethyl octanoate.

Orthogonal partial least-squares discriminant analysis (OPLS-DA) was used to identify the key odorants responsible for the aroma distinction between Xijiao_SCT and Xijiao_LCT. OPLS-DA, an improvement over the PLS-DA approach, uses a single component as a predictor for the class, while the other components describe variation orthogonal to the first predictive component. This approach reduces model complexity, preserves prediction ability, effectively removes non-correlated variation in X, and improves interpretative ability for both correlated and non-correlated variations [38]. The results of the k-fold validation and permutation tests shown in Fig. S4 demonstrate the predictability and reliability of this model. Additionally, the score plot in Fig. 4E shows an observable regional distribution of Xijiao_SCT and Xijiao_LCT. By analyzing the VIP scores plot (Figs. 4F), 13 discriminant compounds were identified that differentiate Xijiao_SCT from Xijiao_LCT. These compounds include 5-methyl-2-phenyl-2-hexenal, ethyl isobutyrate, 2-methyl-2-hexenal, succinic acid, butyl ethyl ester, 2-phenyl-2-butenal, 2-heptyl-1,3-dioxepane, 3-methylbutanal, 2,4,5-trimethyl-1,3-dioxolane, furfural, 2-*t*-butyl-5-methyl-1,3-dioxolan-4-one, hinokitiol, benzeneacetaldehyde, and ethyl isovalerate, which seem to be the potential cellar markers of Xijiao, akin to the ageing markers of MCRW. Notably, some of these compounds, such as 5-methyl-2-phenyl-2-hexenal and 2-phenyl-2-butenal, have a cocoa flavor and were detected in the CRW for the first time (Fig. S5). Previous studies have documented the presence of these substances in cocoa and cocoa products as dehydrated aldol condensation products derived from phenylacetaldehyde and other Strecker aldehydes, including 2-methylpropanal and 3-methylbutanal [39,40]. Although their thresholds have not been described, the high concentrations of these compounds suggest their potential impact on the cocoa aroma in Xijiao_LCT.

## 4 Conclusion

This study used GC-MS and multivariate statistical analyses to investigate the sensory properties of ACRW (Xijiao) with varying cellar periods and key aroma compounds contributing to aroma attributes. The data suggest that Xijiao_SCT has a distinct aroma profile that combines the flavor traits of MCRW (alcoholic and grain-like) and GW (fruity and floral). However, Xijiao_LCT exhibited an intense odor of cocoa and caramel. In total, 128 volatile components were identified using untargeted GC-MS. Alcohols, esters, acids, and aldehydes were the predominant aromatic compounds in all the samples. In Xijiao, alcohols and acids decreased with time, while aldehydes increased during the cellar period. Similar trends were observed in the natural ageing of MCRW and the biological ageing of GW. However, unlike MCRW, the ester content in Xijiao initially increased and then decreased with ageing time. This phenomenon is also observed in the biological ageing process of GW and may contribute to the unique aroma attributes of Xijiao. The in-bottle transformation process of Xijiao shares similarities with the biological ageing of GW due to microbial activity. Hence, the microbiome-mediated ageing in term of biological-ageing-like process may help develop particular flavors in CRW.

By calculating the ROAVs, 27 aroma substances were identified as key odorants that significantly contributed to the aroma profile of Xijiao. Furthermore, through multivariate statistical analyses (PCoA, Spearman's correlation analysis, PLS-DA, and OPLS-DA), 12 key odorants were identified that distinguished Xijiao from the other wines, and 13 markers for biological-ageing-like process were identified that differentiated Xijiao_SCT from Xijiao_LCT. Moreover, two substances were implicated in the formation of the cocoa flavor of Xijiao_LCT. This work elucidates the importance of the microbe-modulated ageing process in the production of CRW. It shows that the microbial communities preserved after fermentation process might be considered as a potentially significant contributor to sensory feature and composition of aroma compounds during the bottle aging, leading to a remarkable modification of a rice wine's chemical signature. Our data not only highlight the unique aroma characteristics of ACRW but also demonstrate the continuation and succession of ACRW through biological-ageing-like process, offering promising potential for industrial applications. However, further research is needed to investigate composition of the microbial community diversifying the metabolites and the latent functional components with health benefits in Xijiao, uncovering the secrets behind ACRW.

## Ethics statement

The sensory evaluation was carried out in accordance with the established ethical guidelines of the Declaration of Helsinki. Verbal informed consent was obtained from every participant after explaining the nature of the study in understandable terms. Participants were provided with a detailed explanation of the study's purpose, procedures, and data usage. They voluntarily agreed to participate by affirming, “I am aware that my responses are confidential, and I agree to participate in this survey.” An affirmative reply was necessary to access the survey. Questionnaires used in the study were provided to participants with clarity and comprehensibility. The methodology employed in this study aimed to minimize potential harm or discomfort to participants while maximizing the validity and reliability of the results. The study was conducted with utmost respect for participants' rights and confidentiality. Any identifiable information collected from participants was anonymized to ensure their privacy. Therefore, we adopted verbal informed consent instead of written informed consent to ensure that the privacy of participants is protected. The products tested were safe for consumption and all the physicochemical indexes meet the criteria according to national standards GB/T 2–1366, 2018. The participants were able to withdraw from the survey at any time without giving a reason.

## Data availability statement

The main data supporting the findings of this study are available within the article and its Supplementary Information.

## CRediT authorship contribution statement

**Han Wang:** Writing – original draft, Visualization, Validation, Methodology, Formal analysis, Data curation. **Rui Shang:** Methodology. **Suying Gao:** Methodology. **Ancheng Huang:** Methodology. **Honghui Huang:** Conceptualization. **Wenyang Li:** Writing – review & editing, Project administration, Investigation, Funding acquisition, Conceptualization. **Hongwei Guo:** Writing – review & editing, Supervision, Resources, Project administration, Funding acquisition, Conceptualization.

## Declaration of competing interest

The authors declare that they have no known competing financial interests or personal relationships that could have appeared to influence the work reported in this paper.
