# Supplementary material for: Characterization of key aroma compounds in a novel Chinese rice wine Xijiao Huojiu during its biological-ageing-like process by untargeted metabolomics
Source: Heliyon. 2024 Jul 10;10(14):e34396. doi: 10.1016/j.heliyon.2024.e34396 (PMC11315155; doi:10.1016/j.heliyon.2024.e34396)
Supplement: Multimedia component 1 [file mmc1.docx]

**Table S1.**

Sensory evaluation questionnaire.

| Attributes | 0 | 0.5 | 1 | 1.5 | 2 | 2.5 | 3 | 3.5 | 4 | 4.5 | 5 |
| --- | --- | --- | --- | --- | --- | --- | --- | --- | --- | --- | --- |
| Alcoholic |  |  |  |  |  |  |  |  |  |  |  |
| Fruity |  |  |  |  |  |  |  |  |  |  |  |
| Floral |  |  |  |  |  |  |  |  |  |  |  |
| Caramel-like |  |  |  |  |  |  |  |  |  |  |  |
| Honey |  |  |  |  |  |  |  |  |  |  |  |
| Sour |  |  |  |  |  |  |  |  |  |  |  |
| Nutty |  |  |  |  |  |  |  |  |  |  |  |
| *Qu* |  |  |  |  |  |  |  |  |  |  |  |
| Creamy |  |  |  |  |  |  |  |  |  |  |  |
| Bitter |  |  |  |  |  |  |  |  |  |  |  |
| Cocoa |  |  |  |  |  |  |  |  |  |  |  |
| Herbal |  |  |  |  |  |  |  |  |  |  |  |
| Grain-like |  |  |  |  |  |  |  |  |  |  |  |
| Woody |  |  |  |  |  |  |  |  |  |  |  |
