# Supplementary material for: Characterization of key aroma compounds in a novel Chinese rice wine Xijiao Huojiu during its biological-ageing-like process by untargeted metabolomics
Source: Heliyon. 2024 Jul 10;10(14):e34396. doi: 10.1016/j.heliyon.2024.e34396 (PMC11315155; doi:10.1016/j.heliyon.2024.e34396)
Supplement: Multimedia component 2 [file mmc2.docx]

**Table S2.**

Relative odor activity values (ROAVs) of aroma compounds in all samples.

| Aroma compound | Threshold (μg/L)^a^ | Odor activity value (OAV) | | | | |  |  |  |  | Odor description |
| --- | --- | --- | --- | --- | --- | --- | --- | --- | --- | --- | --- |
|  |  | YJ | JJ | NEH | GLS | SKM | KJS | XH | CC | ZY |  |
| Acetic acid | 2190.0 | 0.018 | 0.175 | 0.247 | 16.449 | 10.927 | 11.073 | 0.699 | -^b^ | - | Pungent, sour, vinegar-like |
| Decanoic acid | 130.0 | 0.221 | 0.097 | - | - | - | - | - | 0.577 | 2.044 | Unpleasant, rancid, creamy, dust, fat, grass |
| Hexadecanoic acid | 1000.0 | 0.024 | 0.019 | - | - | - | - | - | - | - | Odorless, slight characteristic |
| Nonanoic acid | 1100.0 | 0.010 | 0.009 | - | - | - | - | - | - | - | Waxy, dirty, cheesy, dairy |
| Isovaleric acid | 33.0 | 0.244 | 0.213 | - | 0.791 | 0.188 | 0.510 | 0.373 | 0.190 | 0.358 | Penetrating, disagreeable, rancid, cheesy |
| Hexanoic acid | 806.5 | 0.008 | - | - | - | - | - | - | - | - | Unpleasant, cheesy, sweat-like, characteristic goat-like |
| 2-Methylbutanoic acid | 34.0 | - | 0.160 | 0.194 | 0.161 | 0.141 | - | 0.263 | 0.168 | 0.353 | Butter, cheesy, fermented, sour |
| Octanoic acid | 500.0 | 0.370 | 1.096 | - | - | - | - | - | - | 0.955 | Unpleasant, fruity-acid |
| Phenylethyl alcohol | 250.0 | 4.91 | 10.81 | 9.21 | 27.14 | 27.22 | 36.64 | 24.85 | 17.62 | 39.93 | Rose, honey |
| Isoamyl alcohol | 250.0 | 4.25 | 3.12 | 2.90 | 3.29 | 2.80 | 5.96 | 4.84 | 6.95 | 12.83 | Apple, brandy, spicy, bread, grain |
| 1-Propanol | 10695.2 | 0.943 | 1.819 | 1.067 | 1.376 | - | 0.749 | 1.098 | 0.335 | 0.485 | Mild, alcohol-like |
| Isobutanol | 40000.0 | 0.102 | 0.609 | 0.735 | 3.912 | 2.451 | 4.089 | 0.460 | 0.260 | 0.380 | Penetrating, wine-like, disagreeable |
| 1-Octanol | 110.0 | 0.164 | 0.310 | - | - | - | 0.211 | 0.167 | 0.097 | 0.383 | Penetrating, rose, orange, lemon |
| 2,3-Butanediol | 1800.0 | 0.001 | - | - | - | - | - | 0.027 | - | - | Ripe fruit, buttery |
| 1-Hexanol | 2500.0 | - | - | - | - | - | 0.004 | - | 0.011 | 0.072 | Banana, floral, grass, herb |
| 2-ethylhexan-1-ol | 300.0 | 0.009 | - | - | - | - | - | 0.034 | 0.024 | - | Mild, oily, sweet, rose |
| 2-Methyl-1-butanol | 32000.0 | - | - | - | - | - | - | - | 0.026 | 0.007 | Fish oil, green, malt, onion, wine |
| Benzyl alcohol | 900.0 | - | - | - | - | - | - | - | 0.068 | 0.085 | Boiled cherries, moss, roasted bread, rose |
| Texanol | 580.0 | - | - | - | - | - | - | - | - | 0.018 | Mild, characteristic |
| Ethanol | 10000.0 | 0.429 | 0.311 | 0.282 | 0.750 | 0.821 | 1.161 | 0.661 | 0.964 | 1.034 | Strong alcoholic |
| 2-Nonanol | 58.0 | 0.333 | 1.412 | - | - | - | - | 0.294 | - | 0.203 | Cucumber, green, fatty, melon |
| 1-Nonanol | 34.0 | - | - | - | - | - | - | - | 0.395 | 1.736 | Fat, floral, green, oil |
| Furfural | 3000.0 | 0.008 | 0.160 | 0.722 | 0.547 | 0.519 | 0.558 | 0.359 | 0.009 | 0.023 | Almond, baked potatoes, bread, burnt, caramel, grain |
| 3-Methylbutanal | 120.0 | - | - | 2.481 | - | - | - | - | - | - | Apple, peach, malty |
| 5-Methyl-2-furaldehyde | 16.0 | - | - | 2.518 | - | - | - | - | - | - | Spicy-sweet, warm, caramel, nutty |
| Methacrylaldehyde | 25.0 | - | - | - | 7.773 | 8.245 | 5.641 | - | - | - | Pungent, characteristic |
| 1,1-Diethoxyethane | 100.0 | - | - | - | 0.204 | 0.207 | 0.206 | 0.092 | - | - | Creamy, fruit, pleasant, tropical fruit |
| Nonanal | 35.2 | - | - | - | - | - | 0.750 | - | - | - | Fat, floral, green, lemon |
| Benzaldehyde | 350.0 | 0.018 | 0.175 | 0.247 | 16.449 | 10.927 | 11.073 | 0.699 | - | - | Sweet, cherry, fruity, roasted, caramel, almond, nutty |
| Benzeneacetaldehyde | 4.0 | 0.665 | 12.630 | 48.033 | - | - | 11.683 | 14.330 | - | - | Floral, sweet, hyacinth, chocolate |
| Ethyl acetate | 5.0 | 281.40 | 338.91 | 378.78 | 357.32 | 176.60 | 354.08 | 373.77 | 938.28 | 253.05 | Fruity, pineapple, apple, banana |
| Ethyl octanoate | 5.0 | 231.97 | 344.08 | 66.53 | 54.15 | 36.20 | 63.37 | 238.43 | 556.96 | 255.22 | Fruity, brandy, winey, pineapple, apricot |
| Ethyl L-lactate | 14000.0 | 0.246 | 1.394 | - | - | - | - | - | 0.424 | 0.415 | Rum, fruity, creamy, fatty |
| Ethyl hexadecanoate | 1500.0 | - | - | - | 1.146 | 0.807 | 0.901 | - | - | - | Mild waxy, creamy |
| 2-Octyl acetate | 38.0 | 2.172 | 2.885 | 3.032 | 4.317 | 3.199 | 4.268 | 4.684 | 1.595 | 0.505 | Fruity |
| Isoamyl acetate | 30.0 | 2.389 | 4.881 | 2.780 | 1.160 | 0.617 | 0.591 | 3.639 | 3.143 | 3.025 | Fruity, pear, banana-like, sweet, fragrant |
| Ethyl dodecanoate | 1500.0 | 0.046 | 0.045 | 0.028 | - | - | 0.009 | - | 0.013 | 0.013 | fatty, fruity, floral |
| Phenethyl acetate | 250.0 | 0.189 | 0.671 | 0.338 | 0.263 | 0.293 | 0.587 | 0.826 | 0.378 | 0.702 | Very sweet, rosy, honey |
| Ethyl 2-hydroxy-4-methylpentanoate | 1220.0 | 0.033 | 0.335 | - | 0.077 | 0.045 | 0.305 | 0.067 | 0.010 | 0.025 | Fruity, blue berry, tropical fruit, lime, valerian oil |
| Isoamyl lactate | 3.0 | 11.980 | 133.473 | - | 10.743 | 5.650 | 21.850 | 16.920 | 5.393 | 28.967 | Fruity |
| Ethyl hexanoate | 14.0 | 2.183 | - | - | - | - | - | - | - | - | Fruity, apple peel, brandy, fruit gum, overripe fruit, pineapple |
| Ethyl nonanoate | 200.0 | 0.140 | 0.113 | - | 0.176 | - | 0.122 | - | 0.053 | - | Fruity, grape, flroal, rose, brandy |
| Ethyl Oleate | 870.0 | 0.032 | 0.014 | - | - | - | 0.051 | - | 0.003 | - | Floral |
| Ethyl isobutyrate | 5.6 | 4.138 | 3.914 | 16.379 | - | - | - | 2.820 | 10.168 | - | Fruity, pleasant |
| Ethyl tetradecanoate | 2000.0 | 0.011 | 0.006 | - | - | - | - | - | - | - | Waxy, orris |
| Ethyl linoleate | 450.0 | 0.040 | 0.041 | - | - | - | - | - | - | - | Mild floral, oil |
| Isobutyl acetate | 66.0 | 0.251 | 0.244 | 0.113 | - | - | - | 0.178 | - | - | Fruity, currant, pear, floral, hyacinth, rose |
| Ethyl octadecanoate | 500.0 | 0.015 | 0.018 | - | - | - | - | - | 0.008 | - | Little odor |
| Isobutyl octanoate | 800.0 | 0.005 | 0.012 | - | - | - | - | - | - | - | Fruity, floral, green |
| Ethyl heptanoate | 2.0 | 1.215 | 4.300 | 1.630 | 20.910 | 3.825 | 20.290 | 4.965 | - | - | Pineapple, berry, plum, brandy |
| Ethyl isovalerate | 3.0 | - | 4.287 | 39.380 | 14.677 | 5.227 | - | - | 2.067 | 2.250 | Fruity, vinous, apple |
| Ethyl valerate | 5.0 | - | - | - | 3.584 | - | 3.526 | - | - | - | Apple, dry fish, herb, nut, yeast |
| Ethyl salicylate | 84.0 | - | - | - | 0.194 | - | - | - | - | - | Spicy, anisic, wintergreen |
| gamma-Nonalactone | 65.0 | - | - | - | - | - | 1.246 | - | - | - | Coconut, creamy, waxy, fatty, milky |
| Ethyl 3-phenylpropionate | 70.0 | - | - | - | - | - | 0.622 | - | - | - | Flower, honey |
| Ethyl propanoate | 400.0 | 0.086 | 0.014 | - | - | - | 0.074 | 0.022 | - | - | Apple, pineapple, rum, strawberry |
| 4-Ethoxy-4-oxobutanoic acid | 1000.0 | - | - | - | - | - | - | - | 0.333 | - | Fruity |
| Pentyl acetate | 180.0 | - | - | - | - | - | - | - | 0.261 | - | Banana |
| Isopentyl octanoate | 125.0 | - | - | - | - | - | - | - | 0.161 | - | Fruity |
| cis-3-Methyl-4-octanolide | 35.0 | - | - | - | - | - | - | - | 0.426 | 0.346 | Woody, coconut, vanilla |
| Methyl octanoate | 200.0 | - | - | - | - | - | - | - | 0.018 | - | Fruit, orange, wax, wine |
| delta-Dodecalactone | 53.0 | - | - | - | - | - | - | - | - | 0.199 | Fruity, peach, pear, plum |
| Isoamyl decanoate | 5000.0 | 0.001 | - | - | - | - | - | - | - | - | Waxy, banana, fruity, sweet, green |
| 2-Methylbutyl acetate | 11.0 | 2.304 | - | - | - | - | - | - | - | - | Apple, banana, pear |
| Ethyl decanoate | 200.0 | 2.279 | 2.019 | 1.392 | - | 0.049 | 0.269 | 0.397 | 5.218 | 1.812 | Brandy, grape, pear, coconut, rose |
| Diethyl succinate | 1200.0 | 0.321 | 0.770 | 0.739 | 1.231 | 0.859 | 2.740 | 1.520 | 2.128 | 4.393 | Pleasant, floral, fruity, wine |
| Ethyl butyrate | 20.0 | 0.943 | 1.819 | 1.067 | 1.376 | - | 0.749 | 1.098 | 0.335 | 0.485 | Fruity, pineapple, apple, banana |
| Ethyl phenylacetate | 73.0 | 0.102 | 0.609 | 0.735 | 3.912 | 2.451 | 4.089 | 0.460 | 0.260 | 0.380 | Sweet, pleasant, honey |
| Ethyl 2-methylbutyrate | 18.0 | 0.246 | 1.394 | - | - | - | - | - | 0.424 | 0.415 | Fruity, green apple, kiwi, strawberry |
| gamma-Undecalactone | 4.0 | 0.530 | 1.445 | 3.073 | - | - | 15.075 | - | - | - | Fruity, apricot, peach, nutty, vanilla |
| Ethyl benzoate | 500.0 | - | - | - | 1.146 | 0.807 | 0.901 | - | - | - | Camomile, celery, fat, flower, fruit |
| Furfuryl alcohol | 2000.0 | 0.002 | - | - | - | - | - | - | - | - | Burnt, caramel, cooked |
| Tetradecane | 1000.0 | - | - | - | 0.044 | 0.029 | - | - | - | - | Fragrant |
| Styrene | 100.0 | - | - | - | - | - | 1.163 | - | - | - | Sweet, floral, balsamic |
| m-Xylene | 1000.0 | - | - | - | - | - | 0.077 | - | - | - | Sweet, aromatic |
| O-xylene | 450.2 | - | - | - | - | - | - | - | - | 0.018 | Sweet, aromatic |
| 2-(Ethoxymethyl)furan | 11.0 | 1.910 | 9.181 | 1.096 | - | 1.512 | - | - | 0.577 | 1.585 | Sweet, nutty, spicy |
| 1,1,6-Trimethyl-1,2-dihydronaphthalene | 2.5 | - | - | - | - | - | - | - | 9.232 | 8.612 | Licorice, burned, tobacco, herb, petrol |
| 4-Ethyl-2-methoxyphenol | 6.9 | 3.674 | 12.480 | - | - | - | - | - | 7.678 | - | Sweet, spicy, medicinal, clove |
| 4-Ethylphenol | 51.0 | 0.369 | 1.754 | 0.489 | - | - | - | - | 1.955 | 0.910 | Woody, phenolic, medicinal, sweet |
| 2,4-Di-tert-butylphenol | 500.0 | 0.011 | 0.011 | 0.011 | 0.041 | 0.036 | 0.041 | - | 0.045 | 0.090 | Characteristic, alkyl phenol-like |
| Butylated Hydroxytoluene | 1000.0 | - | - | - | - | - | - | 0.015 | - | - | Musty, cresylic-like, toasted cereal |
| 2-Octanone | 1100.0 | 0.094 | 0.231 | 0.316 | 0.166 | 0.169 | 0.164 | 0.240 | 0.081 | 0.052 | Fruity, apple, floral, fatty, cheesy |
| 3-Nonen-2-one | 800.0 | - | - | 0.014 | - | - | - | - | - | - | Fruity, berry, wet |
| Acetophenone | 65.0 | - | - | - | 0.863 | 0.256 | 0.469 | - | - | - | Almonds, flower, meat, must |
| 2-Heptanone | 24.0 | - | - | - | 0.428 | - | - | - | - | - | Blue cheese, fruit, green, nut, spice |
| 2-Nonanone | 75.0 | - | - | - | - | - | 0.366 | - | - | - | Fragrant, fruit, green, hot milk |
| alpha-Ionone | 8.0 | - | - | - | - | - | - | - | 20.290 | 12.683 | Sweet, floral, violets, woody |
| Linalool | 6.0 | - | - | - | - | - | - | - | - | 2.157 | Coriander, floral, lavender, lemon, rose |

^a^ Odor threshold values were obtained from the book *Compilations of Odour Threshold Values in Air, Water & other Media*.

^b^ “-” represents “not detected”.
