# Supplementary material for: Characterization of key aroma compounds in a novel Chinese rice wine Xijiao Huojiu during its biological-ageing-like process by untargeted metabolomics
Source: Heliyon. 2024 Jul 10;10(14):e34396. doi: 10.1016/j.heliyon.2024.e34396 (PMC11315155; doi:10.1016/j.heliyon.2024.e34396)
Supplement: Multimedia component 3 [file mmc3.docx]

**Fig. S1. Physicochemical indicators of *Xijiao Huojiu*.**


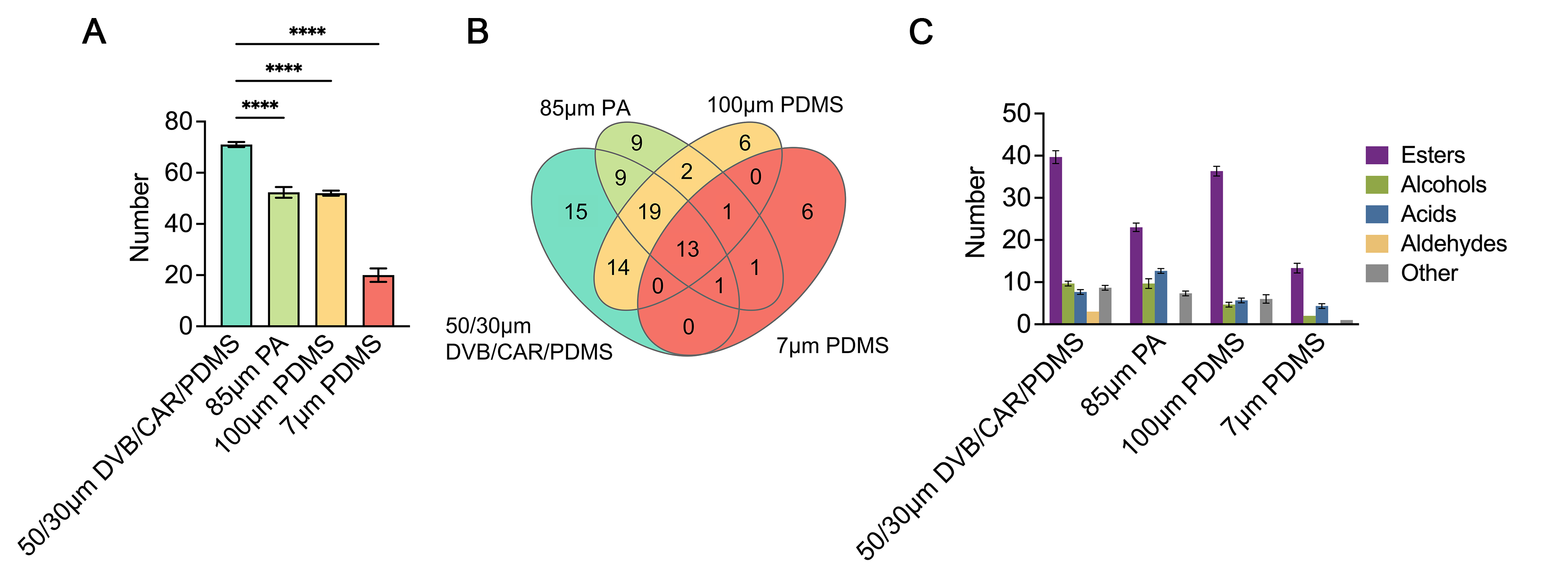


**Fig. S2. Comparison of extraction capacity of four candidate solid phase microextraction (SPME) fibers.**


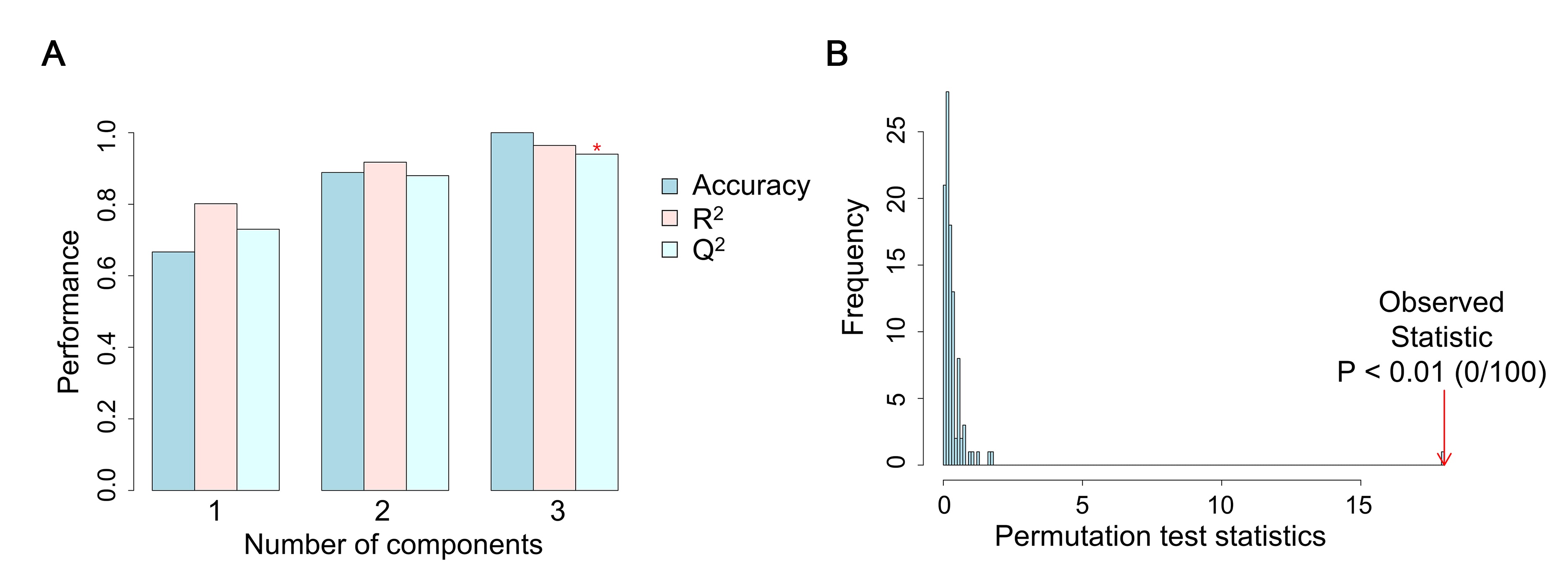


**Fig. S3.** Validation of partial least squares-discriminant analysis (PLS-DA) model.


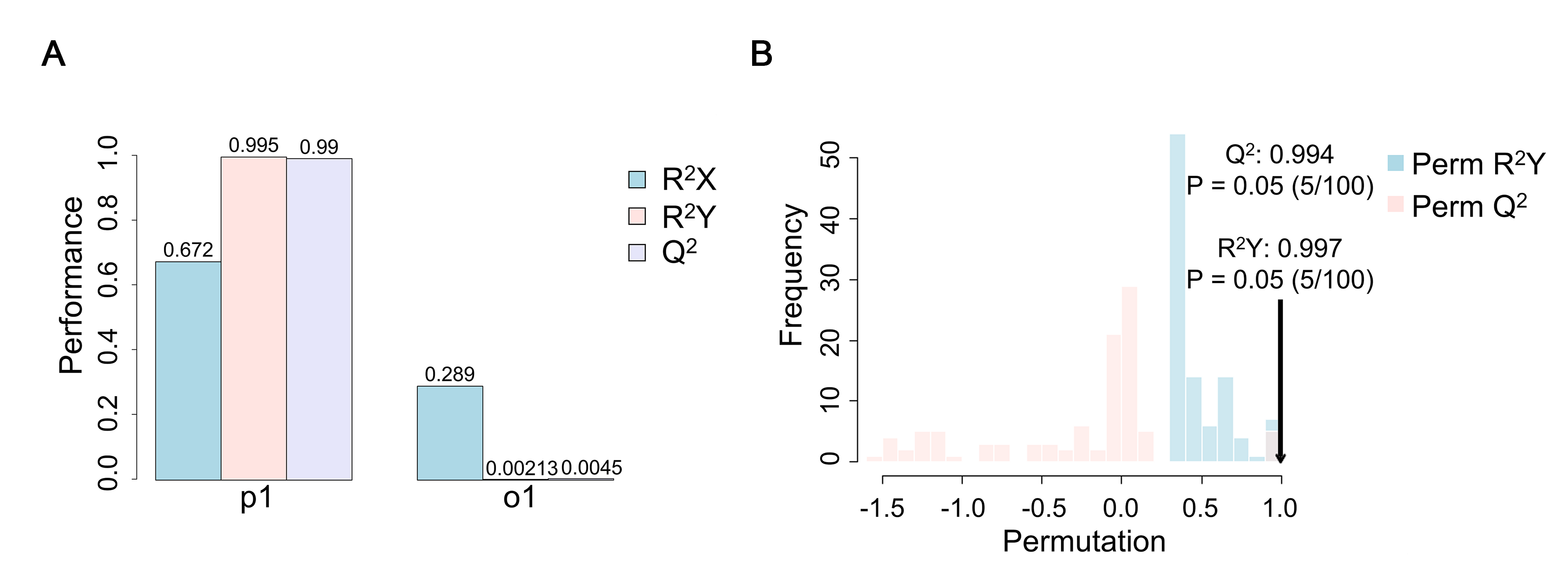


**Fig. S4.** Validation of orthogonal partial least-squares discriminant analysis (OPLS-DA) model.


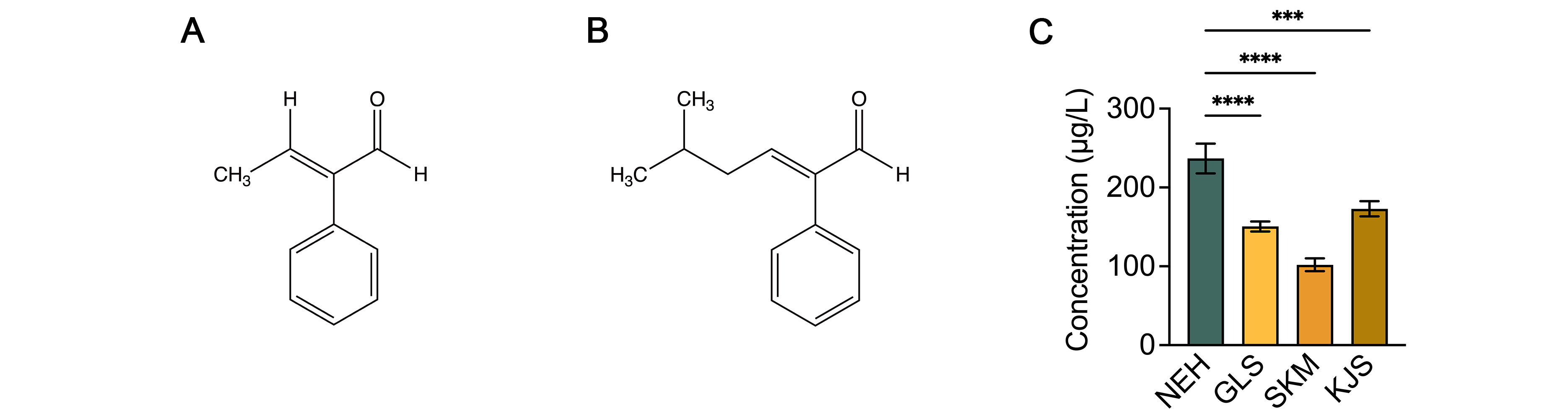


**Fig. S5.** Two potential aroma compounds that may participate in the development of cocoa flavor in Xijiao_LCT.
